# The genomes of *Vischeria* oleaginous microalgae shed light on the molecular basis of hyper-accumulation of lipids

**DOI:** 10.1186/s12915-023-01618-x

**Published:** 2023-06-06

**Authors:** Baoyan Gao, Meng Xu, Dai Shan, Chi Zhang, Yulan Yang, Zhensheng Dong, Hu Zhang, Boping Han, Luodong Huang, Chengwu Zhang

**Affiliations:** 1 Department of Ecology & Research Center for Hydrobiology, Jinan University, Guangzhou, 510632 China; 2 BGI Genomics, BGI-Shenzhen, Shenzhen, China

**Keywords:** *Vischeria* sp. CAUP H4302, *Vischeria stellata*, Genome analysis, Whole-genome duplication, Triacylglycerols, Cyanate lyase

## Abstract

**Background:**

With the urgent need to reduce carbon emissions, and the dwindling reserves of easily exploitable fossil fuel, microalgae-based biofuels that can be used for transport systems and CO_2_ abatement have attracted great attention worldwide in recent years. One useful characteristic of microalgae is their ability to accumulate high levels of lipid content, in particular under conditions of nitrogen deprivation, with numerous species identified so far. However, a trade-off between levels of lipid accumulation and biomass productivity hinders the commercial applicability of lipids from microalgae. Here, we sequenced the genomes of *Vischeria* sp. CAUP H4302 and *Vischeria stellata* SAG 33.83, which can accumulate high content of lipids rich in nutraceutical fatty acids and with excellent biomass yield in nitrogen-limiting culture.

**Results:**

A whole-genome duplication (WGD) event was revealed in *V*. sp. CAUP H4302, which is a rare event in unicellular microalgae. Comparative genomic analyses showed that a battery of genes encoding pivotal enzymes involved in fatty acids and triacylglycerol biosynthesis, storage polysaccharide hydrolysis, and nitrogen and amino acid-related metabolisms are expanded in the genus *Vischeria* or only in *V*. sp. CAUP H4302. The most highlighted is the expansion of cyanate lyase genes in the genus *Vischeria*, which may enhance their detoxification ability against the toxic cyanate by decomposing cyanate to NH_3_ and CO_2_, especially under nitrogen-limiting conditions, resulting in better growth performance and sustained accumulation of biomass under the aforementioned stress conditions.

**Conclusions:**

This study presents a WGD event in microalgae, providing new insights into the genetic and regulatory mechanism underpinning hyper-accumulation of lipids and offering potentially valuable targets for future improvements in oleaginous microalgae by metabolic engineering.

**Supplementary Information:**

The online version contains supplementary material available at 10.1186/s12915-023-01618-x.

## Background

As a green cell factory for CO_2_ sequestration, photosynthetic microalgae have been considered as a promising biofuel feedstock providing sustainable bioenergy with many advantages, such as the high photosynthetic efficiency of carbon fixation and the ability to massively accumulate lipid substances as energy and carbon reserves [1]. Researchers have found a number of oleaginous microalgae with more than 50% lipid content of dry weight (DW). However, there is an inverse relationship between lipid content and biomass productivity in microalgae [2], namely, biomass accumulation is markedly retarded under nitrogen-limiting conditions that favor high lipid accumulation and result in lower lipid productivity [3]. The trade-off between biomass production and lipid accumulation is a main obstacle to restricting the commercial production of lipid from microalgae.

Apparently, prospecting novel oleaginous microalgae strains which can simultaneously accumulate high concentration of biomass and lipid under nitrogen-limited condition is one of appropriate strategies. Furthermore, in order to develop and exploit these novel oleaginous microalgae, understanding the intrinsic molecular basis of their high lipid productivity at the genomic level is a top priority. Therefore, many oleaginous microalgae have been sequenced and analyzed by omics methods to investigate the mechanisms underlying high lipid yields, so as to provide novel candidate targets for genetic engineering to improve lipid productivity [4]. Up to the present, no fewer than twelve complete genomes (twelve strains/versions of six species) of *Nannochloropsis* and *Microchloropsis*, in the family Monodopsidaceae of the order Eustigmatales, which are two well-known genera for high capacity of lipid accumulation and belongs to the Eustigmatophyceae, have been published [5–13]. However, many details about the regulation of central carbon metabolism and the carbon flux distribution remain unclear, especially the molecular mechanisms of high lipid accumulation under nitrogen-limiting conditions. The lack of available genomes of microalgal strains that are capable to achieve high biomass yield under nitrogen deficiency conditions has restricted further research aimed at promoting microalgae-based lipid productivity.

In our previous research, we found that oleaginous microalgae *Vischeria* sp. CAUP H4302 (originally named as *Eustigmatos* cf. *polyphem* obtained from Culture Collection of Algae of Charles University in Prague, and renamed here according to Kryvenda et al. [14]) and *Vischeria stellata* SAG 33.83 obtained from Culture Collection of Algae at the University of Göttingen, which are members of the family Chlorobotryaceae, sister to Monodopsidaceae [15], also in the order Eustigmatales of Eustigmatophyceae, have superior performances in lipid and biomass accumulation under the same nitrogen-limiting conditions [16–18]. In nitrogen-limiting conditions (1 mM of initial nitrogen concentrations (INC) in mBG-11 culture medium), a biomass of 1.68 g/L and lipid content of 58.46% of DW were obtained in *N. oculata.* In contrast, *V. stellata* (*Vischeria stellata* SAG 33.83) and *V.* sp. H4302 (*Vischeria* sp. CAUP H4302) could achieve much higher concentration of biomass of 3.30 and 4.72 g/L and much higher lipid contents of 66.79 and 71.45% of DW, respectively [19]. In addition, the most predominant fatty acid in these two microalgae is palmitoleic acid (POA, > 50% of total fatty acids, > 25% of dry weight). POA is an omega-7 monounsaturated fatty acid and is known to exhibit multiple biological functions and health benefits, such as alleviating the effects of chronic diseases (e.g., obesity, diabetes, cardiovascular diseases) on human health and anti-bacterial activity [20]. The hyper-accumulation of POA makes *V*. sp. H4302 and *V*. *stellata* promising production feedstocks of this bioactive fatty acid.

In this study, we sequenced and assembled two high-quality genomes of *V. stellata* and *V.* sp. H4302. Through comparative genomic and transcriptomic analysis, especially with attention to genes related to lipid metabolism, carbohydrate metabolism, nitrogen metabolism, and photosynthesis process, we investigated the potential molecular mechanisms underlying the stronger innate lipid and POA accumulation ability and high biomass production performance of *V.* sp. H4302 and *V*. *stellata* to reveal their intrinsic specific genetic basis and novel biological characteristics*.*

## Results and discussion

### Genome assemblies and annotation

We sequenced the genome of *V.* sp. H4302 using long-read sequencing technology (PacBio RSII platform) and high-throughput chromosome conformation capture (Hi-C) technology. 19.5 Gb long-read (Additional file 1: Table S1) data was generated and was used in contig assembly. The initial assembly was 229.8 Mb with contig N50 of 680.4 kb (Additional file 1: Table S2). These contigs were clustered and assembled into chromosomes with the aid of 59.1 Gb Hi-C sequencing data (Additional file 1: Table S3). As a result, 224.9 Mb (97.9%) of contigs were anchored into 60 chromosomes (Additional file 1: Fig. S1) with length from 1.55 to 5.39 Mb. The genome of *V. stellata* was sequenced using the PacBio Sequel platform and Hi-C technology, yielding 8.7 and 47.2 Gb data respectively. The contig assembly of *V. stellata* comprised 115.4 Mb with Contig N50 of 1.09 Mb (Additional file 1: Table S4), and 111.4 Mb (96.6%) contigs were assembled into 30 chromosomes (Additional file 1: Fig. S2) with length from 2.61 to 5.19 Mb. The BUSCO [21] evaluation showed a higher percentage of completeness score in these two assemblies (90 and 92%) than in most published algal genomes (59–92%, Additional file 1: Table S5), indicating that these two genome assemblies in this study are complete.

The repetitive elements (REs) represented 51.8 and 34.8% of the genome in *V.* sp. H4302 and *V. stellata*, respectively. Another published genome of *Vischeria* alga (*V.* sp. C74) [22] has a size of 106.5 Mb and contains 47.7% of the REs (Additional file 1: Table S6). In contrast, the RE content was only 1.9–8.7% in species of *Microchloropsis* and *Nannochloropsis* with genome size from 26.9 to 35.5 Mb [5–8] (Additional file 1: Table S6). The expansion of REs may be one important factor contributing to the larger genome size of the three *Vischeria* algae. Subsequently, 18,746 and 12,854 genes were predicted with 91 and 94% BUSCO completeness score (Additional file 1: Table S7) in the genomes of *V.* sp. H4302 and *V. stellata*, respectively. Interestingly, *V*. sp. H4302 contains 62% complete duplicated BUSCO genes, which is much higher than other eustigmatophycean microalgae (1 ~ 3%, Additional file 1: Table S7). Such a high percentage of near-universal gene duplications is hardly explained by RE expansion, suggesting that a whole-genome duplication (WGD) event may have happened in *V*. sp. H4302*.*

### Genome evolution in Eustigmatophyceae and WGD in *V. *sp. H4302

**Fig. 1:**
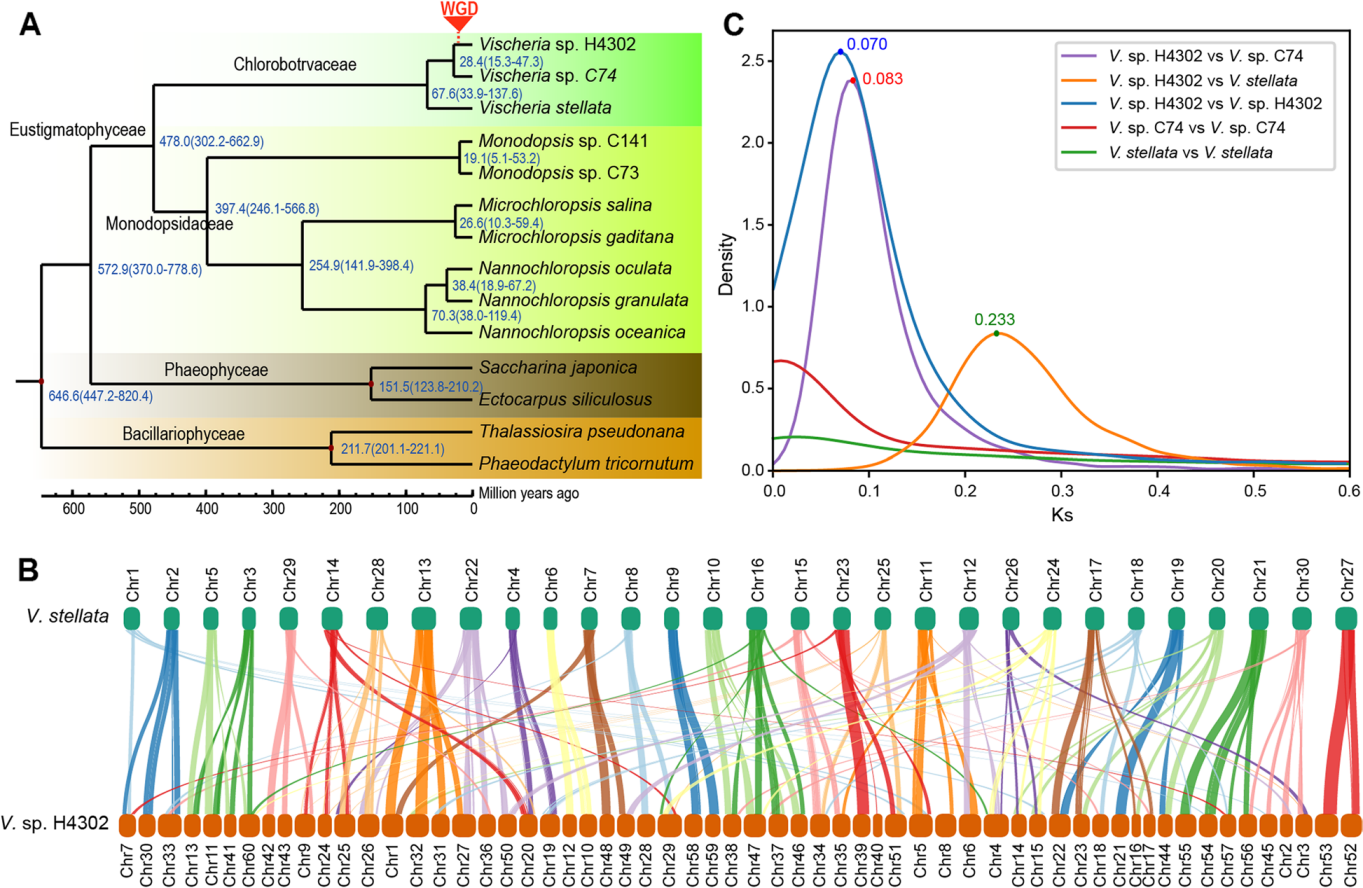
Evolution of two sequenced microalgae and lineage-specific WGD in *V.* cf. *polyphem.***A** Phylogenetic tree and estimated divergence time of 14 species of heterokontophytes. **B** The collinear orthologous blocks (≥ 10 gene pairs) between the genomes of *V.* cf. *polyphem* and *V. stellata.***C** The intragenomic and intergenomic Ks distributions of *V.* cf. *polyphem*, *V.* sp. C74, and *V. stellata*

To explore the genomic evolution of two *Vischeria* algae, we compared the two assemblies with twelve genomes, including eight genomes of Eustigmatophyceae (one of genus *Vischeria*, two of genus *Monodopsis*, and five of family Monodopsidaceae) and four non-Eustigmatophyceae genomes of Heterokontophyta (detailed species see Additional file 1: Table S8). The phylogenetic analysis showed that *V.* sp. H4302 is closer to *V.* sp. C74 than *V. stellata* (Fig. 1A). Based on Bayesian relaxed-molecular clock method, we estimated the divergence time between *V*. sp. H4302 and *V.* sp. C74 to be approximately 28.4 million years ago, and the divergence time between *V*. sp. H4302 and *V*. *stellata* to be approximately 67.6 million years ago (Fig. 1A). To further study the potential WGD event in *V.* sp. H4302, we identified the orthologous genes between *V.* sp. H4302 and the two other *Vischeria* algae, and the paralogous genes within the three algae. We found that most of syntenic orthologous blocks (≥ 10 orthologous genes) showed a ratio of 2:1 of *V.* sp. H4302 and *V*. *stellata* (Fig. 1B). In addition, no paralog peak was presented in *V.* sp. C74 and *V*. *stellata* genome, while a remarkable paralog peak was presented in *V.* sp. H4302 and 93% (5528 out of 5944) of the paralogous gene groups contained two genes (Fig. 1C, Additional file 1: Fig. S3, S4). These results illustrated that one WGD event occurred in *V.* sp. H4302. The synonymous substitution rate (Ks) distribution peak of WGD in *V.* sp. H4302 was about 0.07, and the divergence peak between *V.* sp. H4302 and *V.* sp. C74 and *V*. *stellata* was about 0.083 and 0.233 respectively (Fig. 1B), which implied the WGD event might have occurred about 20.31–23.95 (67.6*0.07/0.233–28.4*0.07/0.083) million years ago.

Doubling of genes provides more materials for evolution. Here, we classified the 5528 paralogous gene pairs into three categories according to the nonsynonymous/synonymous substitution rate ratio (Ka/Ks): stable (< 10%), middle (10 ~ 90%), and dynamic (> 90%) (Additional file 1: Fig. S5A). The dynamic gene pairs have a stronger expression bias (Additional file 1: Fig. S5B), implying that these sister genes have a higher potential to evolve subfunctionalization or neofunctionalization. The stable paralogs have a remarkable more genes in KEGG categories of “transcription” and “translation” [23] than dynamic paralogs (Additional file 1: Fig. S5C), indicating the stabilization in transcription and translation function is important for *V.* sp. H4302 after its WGD event. In contrast, the dynamic paralogs have more genes in “environmental adaptation,” “lipid metabolism,” “glycan biosynthesis and metabolism,” and “metabolism of cofactors and vitamins” categories, indicating the WGD event may facilitate the adaptive evolution and metabolic innovation of some biochemical molecules in *V.* sp. H4302.

**Fig. 2:**
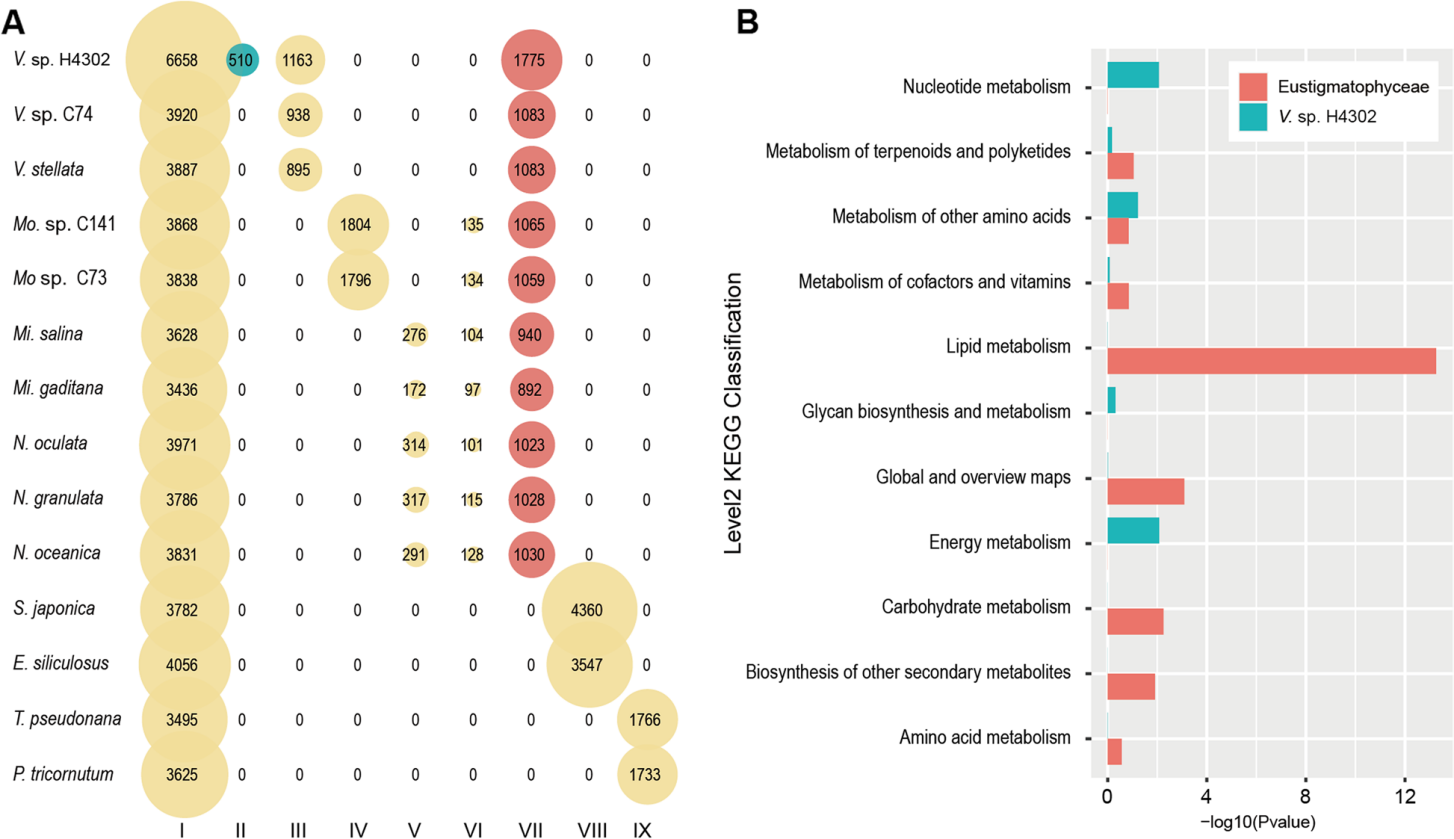
Gene cluster among 14 species of heterokontophytes. **A** Gene number in the corresponding overlapping and specific gene clusters. Column I ~ IX: clusters of genes universal in heterokontophytes. Column II ~ IX: lineage-specific clusters in *V.* sp. H4302, genus *Vischeria*, genus *Monodopsis*, genera *Microchloropsis* + *Nannochloropsis*, Monodopsidaceae, Eustigmatophyceae, Phaeophyceae, and Bacillariophyceae respectively. The lineage-specific clusters of Eustigmatophyceae and *V.* cf. *polyphem* are highlighted with red and green respectively. **B** The functional enrichment of metabolic pathways as the Level 2 KEGG classification for unique genes of Eustigmatophyceae and *V.* cf. *polyphem*. The fill color is in accordance with the fill color highlighted in panel A

A large number of species of the class Eustigmatophyceae are known for their high lipid content [19, 24, 25], and *V.* sp. H4302 can achieve the highest lipid content, reaching 71.45% of DW [19]. To explore the genomic evolution concomitant with the increase of lipid content, we identified lineage-specific genes in Eustigmatophyceae and *V.* sp. H4302, respectively (Fig. 2A). We found that “lipid metabolism” is the most significantly enriched KEGG class (Fig. 2B and Additional file 1: Table S9) for the specific genes in Eustigmatophyceae*.* This may be part of the genomic basis for the altered lipid accumulation ability in Eustigmatophyceae. For the specific genes in *V.* sp. H4302, the top significantly enriched pathway is “Photosynthesis” (Additional file 1: Table S10). In addition, the top 10 enriched pathways also included pathway of “Nitrogen metabolism,” which belongs to “Energy metabolism” at level 2 class, and nitrogen is an essential element for amino acid synthesis. These results provide clues for *V.* sp. H4302 to achieve high biomass yield, especially under nitrogen-limited conditions. It is also noteworthy that two of the top 20 enriched pathways are related to “replication and repair,” which may be associated with the adaption after the WGD event in *V.* sp. H4302.

### Higher POA and TAG synthetic capacity

**Fig. 3:**
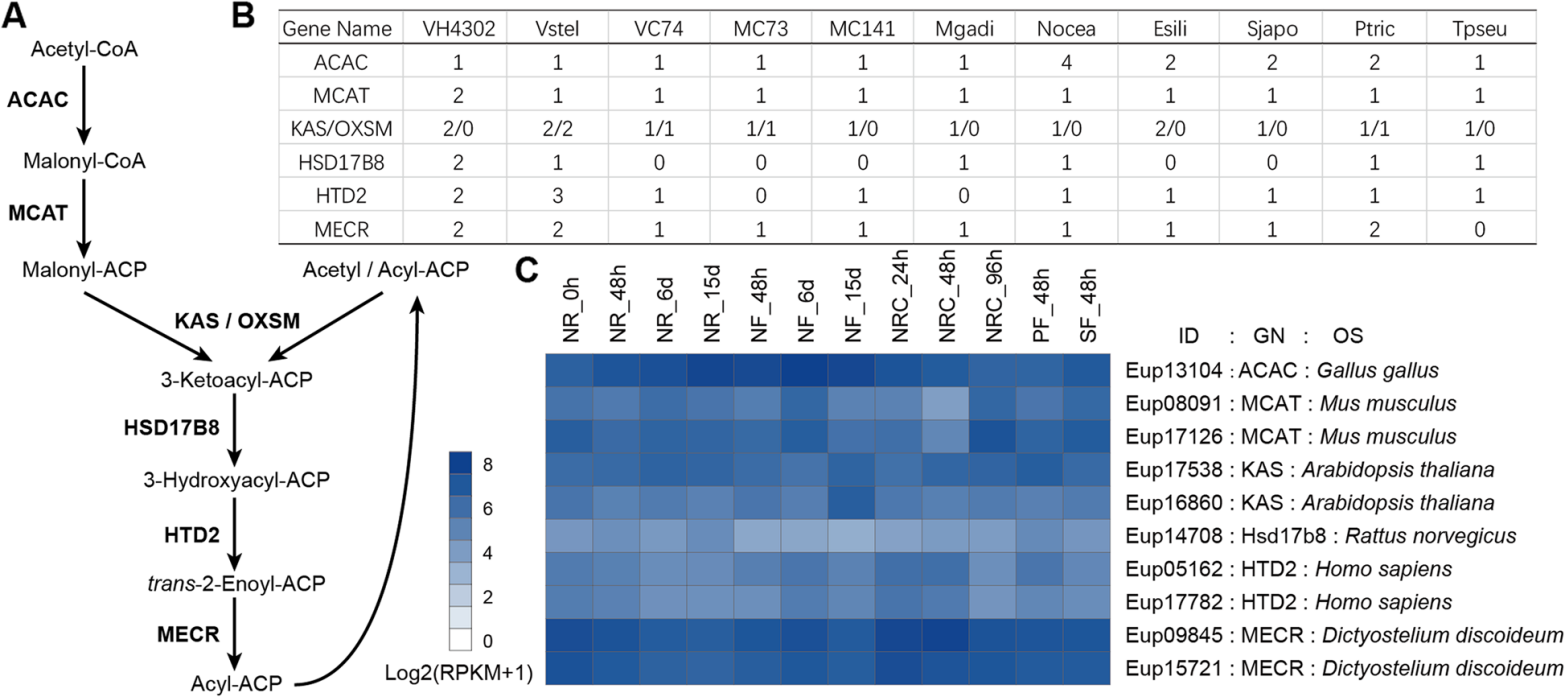
Mitochondrial FAS genes annotation and RNA expression. **A** Schematic overview of the mitochondrial FAS pathway. **B** The numbers of gene copies of mitochondrial FAS genes in eleven species. Abbreviations: Vcfp, *V.* cf. *polyphem*; Vstel, *V. stellata*; VC74, *V.* sp. C74; MC73, *Mo.* sp. C73; MC141, *Mo.* sp. C141; Mgadi, *M. gaditana*; Nocea, *N. oceanica*; Esili, *E. siliculosus*; Sjapo, *S. japonica*; Ptric, *P. tricornutum*; Tpseu, *T. pseudonana*. **C** RNA expression of mitochondrial FAS genes in *V.* cf. *polyphem*. Abbreviations: NR, nitrogen repletion, initial nitrogen concentrations (INC) 18 mM; NF, nitrogen free, INC 0 mM; NRC, nitrogen recovery, 15-day NF cultures were transferred into 18 mM of INC medium; PF, phosphorus free; SF, sulfur-free; ID, gene ID; GN, gene name; OS, the organism species of the best hit sequence belonging in the Swiss-Prot database

Triacylglycerols (TAGs) are the predominant storage lipids in oleaginous species of Eustigmatophyceae [26]. In general, their synthesis includes two steps: fatty acid synthesis (FAS) and glycerolipid synthesis (GLS). In plants and algae, there are two FAS pathways located in plastid (ptFAS) and mitochondria (mtFAS), respectively [27, 28]. The ptFAS pathway is predominant in plants and has been well studied [29], while the identifications of plant mtFAS genes are relatively rare [28]. Here, we identified the components of these two systems and found that all members were present in the genome of *V.* sp. H4302 (Fig. 3A, B, Additional file 1: Fig. S6). The mtFAS genes were expressed during the whole culture of *V.* sp. H4302 (Fig. 3C), indicating that the mitochondrial FAS reactions were active throughout the life cycle in *V*. sp. H4302. Although there was a WGD event in *V.* sp. H4302, only 1 out of 7 ptFAS and 2 out of 6 mtFAS genes have the highest number of copies in *V.* sp. H4302 compared to the other 10 algae. This is not accordant with the situation that *V.* sp. H4302 has the highest lipid content, implying that the FAS is not the rate-limiting process or not the dominant factor of the lipid accumulation in *V.* sp. H4302. The delta-9 desaturase (*FAD9*) catalyze the conversion of palmitic acid (C16:0) to POA. This gene experienced one duplication in the ancestor of *V*. sp. H4302 and *V*. *stellata* (Additional file 1: Fig. S7A). In *V.* sp. H4302, five of the six *FAD9* genes exhibited higher expression in late culture stage or under nutrient (N, P, or S) limiting conditions (Additional file 1: Fig. S7B), which is consistent with the lipid accumulation pattern. The expansion of *FAD9* genes could be associated with high POA content of *V.* sp. H4302 and *V. stellata.*

**Fig. 4:**
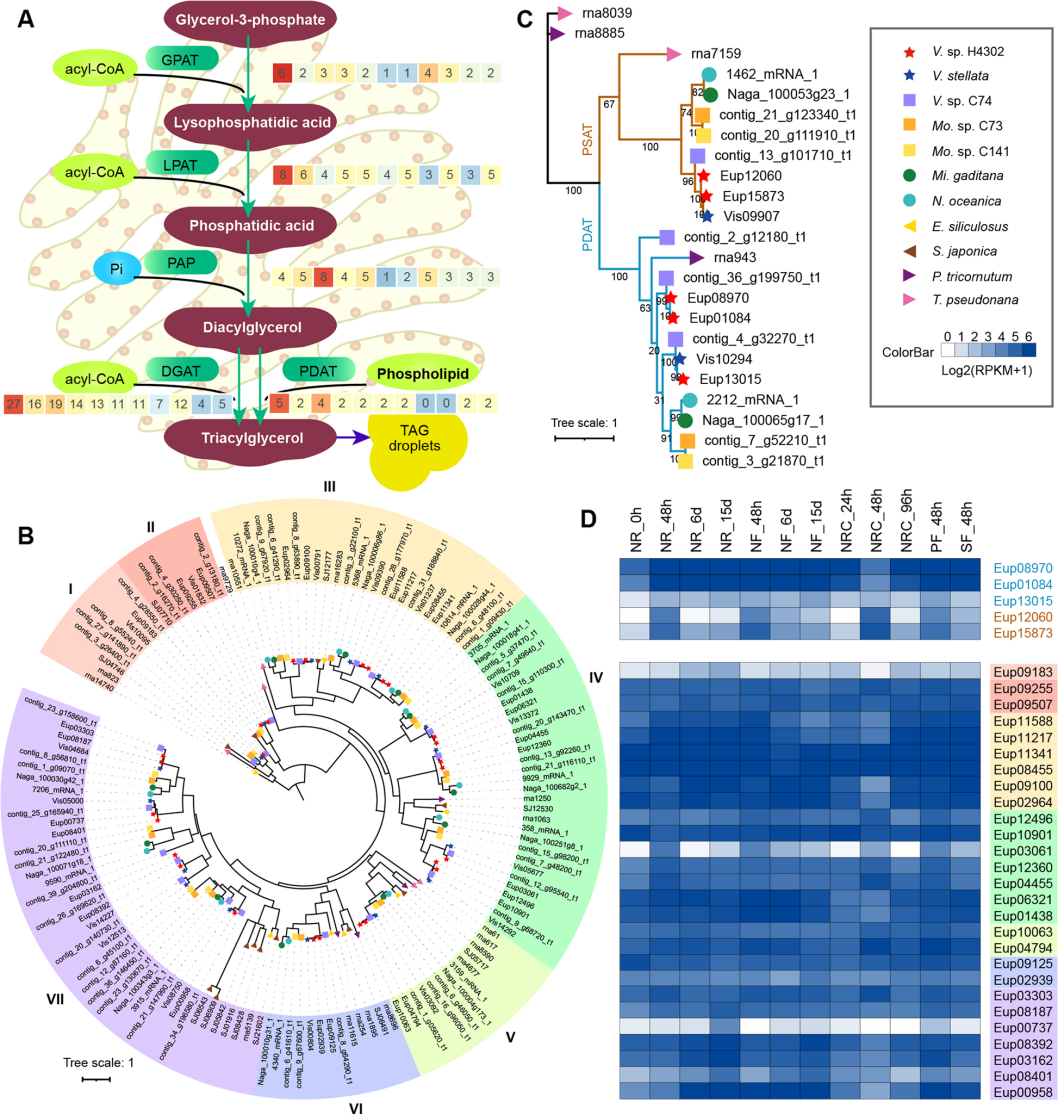
The evolution and RNA expression of two key enzymes in TAG synthesis. **A** Overview of the synthesis pathway from glycerol-3-phosphate to TAG. The gene number in the two sequenced microalgae and nine other heterokontophytes is shown in rectangle (from left to right, *V.* sp. H4302, *V. stellata*, *V.* sp. C74, *Mo.* sp. C73, *Mo.* sp. C141, *Mi. gaditana*, *N. oceanica*, *E. siliculosus*, *S. japonica*, *P. tricornutum*, *T. pseudonana*). The color of rectangle is reflection of relative size of the gene number in each row. Genes: GPAT, glycerol-3-phosphate acyltransferase; LPAT, 1-acyl-sn-glycerol-3-phosphate acyltransferase; PAP, phosphatidic acid phosphatase. (**B**) Phylogenetic tree of the *DGAT* gene family (Pfam motif: PF03982) from eleven heterokont genomes. Seven clades are identified based on the divergence distance and outgroup species (four non-Eustigmatophyceae species). **C** Phylogenetic tree of the *PDAT* gene family (Pfam motif: PF02450). **D** RNA expression of the *PDAT* gene family (upper panel) and the *DGAT* gene family (bottom panel) in *V.* cf. *polyphem*. NR, nitrogen repletion, 18 mM of initial nitrogen concentration (INC); NF, nitrogen-free, 0 mM of INC; NRC, nitrogen recovery, 15 days of NF culture, followed by transfer to 18 mM of INC medium; PF, phosphorus-free; SF, sulfur-free

The second step of TAG synthesis is that GLS uses glycerol-3-phosphate as the skeleton, and incorporates three acyl groups ordinally to form TAGs. According to the donor of the third acyl, TAG synthesis is classified into two pathways: the Kennedy pathway (also known as acyl-CoA-dependent pathway), which is catalyzed by acyl-CoA:diacylglcerol acyltransferase (DGAT), and the acyl-CoA-independent pathway, where a phospholipid is used as acyl donor and which is catalyzed by phospholipid:diacylglcerol acyltransferase (PDAT) (Fig. 4A). The Kennedy pathway is the main pathway in microalgal cells to synthesize TAGs, and DGAT is the key rate-limiting enzyme [30, 31]. Overviewing the gene number distribution of the TAG synthesis genes (Fig. 4A), we found *DGAT* genes had the largest variation in copy number with an incremental expansion pattern from the Heterokontophyta, to the class Eustigmatophyceae, and the genus *Vischeria*. The phylogenetic tree showed that six clades contained at least three of the four non-eustigmatophycean outgroup species, indicating that the ancestor of Heterokontophyta should have had six DGATs (or seven, uncertain for sub-clade II, Fig. 4B). Likewise, based on the four non-eustigmatophycean outgroup species, we could infer that the ancestor of Eustigmatophyceae obtained six extra copies, i.e., two in Clade III, two in Clade IV, and two in Clade VII. The genus *Vischeria* further underwent two other expansion events compared to the Monodopsidaceae, with an additional copy in Clade IV and two copies in clade VII respectively. RNA expression of all 27 copies could be detected in *V.* sp. H4302, and the expression of most *DGAT*s was at similar levels during the whole cultivation process (Fig. 4D), suggesting that the Kennedy pathway consistently remained active and that expanded *DGAT*s contribute to the synthesis of TAGs. The acyl-CoA-independent pathway has overlapping and complementary functions to the Kennedy pathway. There was one copy of *PDAT* in *V. stellata* and other heterokontophytes for comparison, whereas three copies were found in *V.* sp. H4302 and *V.* sp. C74 (Fig. 4C). The *PDAT*s of *V.* sp. H4302 and *V.* sp. C74 shared two distal clades, indicating that the gene duplicated event happened before speciation of two algae and *V.* sp. H4302 lost one copy after its WGD event. Two *PDAT*s (Eup01084 and Eup08970) in the diverged clade of *V.* sp. H4302 presented higher expression in late cultivation period (Fig. 4D), implying the expanded *PDAT*s in *V.* sp. H4302 contributed more to its TAG synthesis in late culture than the non-expanded members. They increased 8.7- and 6.3-fold on the 15th day under nitrogen repletion (NR) conditions, and increased 11.4- and 7.4-fold on the 15th day under nitrogen-free (NF) conditions, but decreased 15.3- and 11.6-fold on the 1st day under nitrogen recovery (NRC) conditions compared to the 15th day of NF conditions, respectively. Previous studies revealed the expansion of *DGAT* genes in Monodopsidaceae, and overexpression of five DGATs increased lipid content and productivity in *N. oceanica* [32]*.* In *Arabidopsis*, it has been revealed that PDAT functions in TAG synthesis. *Dgat1-1* null mutants only have a 20% to 40% decrease in seed oil content, whereas silencing *PDAT1* in the *dgat1-1* null mutant background or silencing *DGAT1-1* in the *pdat1* null mutant background resulted in a 70 to 80% decrease (double mutant of *dgat1-1* and *pdat1* resulted in sterile pollen) [33]. In summary, the evolutionary increase of the copy number of *DGAT* genes in the class Eustigmatophyceae and the genus *Vischeria* may be one genomic factor contributing to the gradual increasing in their TAG synthesis capacity, and the expansion of *PDAT* genes in *V.* sp. H4302 may further improve its TAG accumulation ability.

### Low content of storage polysaccharides

In addition to the synthesis of fatty acids, the fixed carbon dioxide is also used for the synthesis of sugars [34]. It has been reported that the main storage polysaccharide is β-1,3-glucan (also known as chrysolaminarin or laminarin) in Heterokontophyta [35–37]. Through glycosyl residue composition analysis, Vogler et al. reported that chrysolaminarin is the main storage polysaccharide of *M. gaditana* [38]. However, chrysolaminarin could not be efficiently extracted from *V.* sp. H4302 and *V. stellata* using dilute acid method. The reason may be that these two microalgae mainly transiently synthesize chrysolaminarin, rather than storing chrysolaminarin intracellularly. Briefly, chrysolaminarin synthesis starts with glyceraldehyde-3-phosphate, which is converted into glucose, uridine diphosphate glucose (UDPG), linear β-1,3-glucan, and finally branched β-1,3-glucan (chrysolaminarin) [35, 39]. The enzyme β-1,3-glucan synthase (GS) catalyzes the transfer of glucose from UDPG to linear β-1,3-glucan, which is an essential enzyme for chrysolaminarin synthesis [40]. We found three or four copies of *GS* genes in Phaeophyceae, while Eustigmatophyceae have only one or two copies (Additional file 1: Fig. S8), indicating that the chrysolaminarin synthesis ability may be relatively weak in Eustigmatophyceae. On the other hand, the *β-1,3-glucanase* gene (glycosyl hydrolase family 16), which can hydrolyze chrysolaminarin into glucose or glucose derivatives [41], underwent expansion events in *Monodopsis* and *Vischeria*. Both *Mi. gaditana* and *N. oceanica* have two copies, while there are 8–13 copies in species of genus *Monodopsis* and *Vischeria* (Additional file 1: Fig. S9A). *Monodopsis* is closer to *Microchloropsis* and *Nannochloropsis*, so the duplication events in two genera should be independent. The phylogenetic tree shows that there are multiple *β-1,3-glucanase* genes from *Monodopsis* and *Vischeria* clustered together (Additional file 1: Fig. S9A), supporting the speculation of independent duplications. The RNA expression could be detected for ten of these eleven copies in *V.* sp. H4302, and two newly expanded copies showed more than tenfold higher expression than the others (Additional file 1: Fig. S9B). The magnitude of the improved hydrolytic ability may be greater than the difference reflected by the copy number. The expansion of *β-1,3-glucanase* genes may be another evolutionary factor that directs the carbon flux into lipid synthesis.

### Higher biomass yield in nitrogen-limiting conditions

**Fig. 5:**
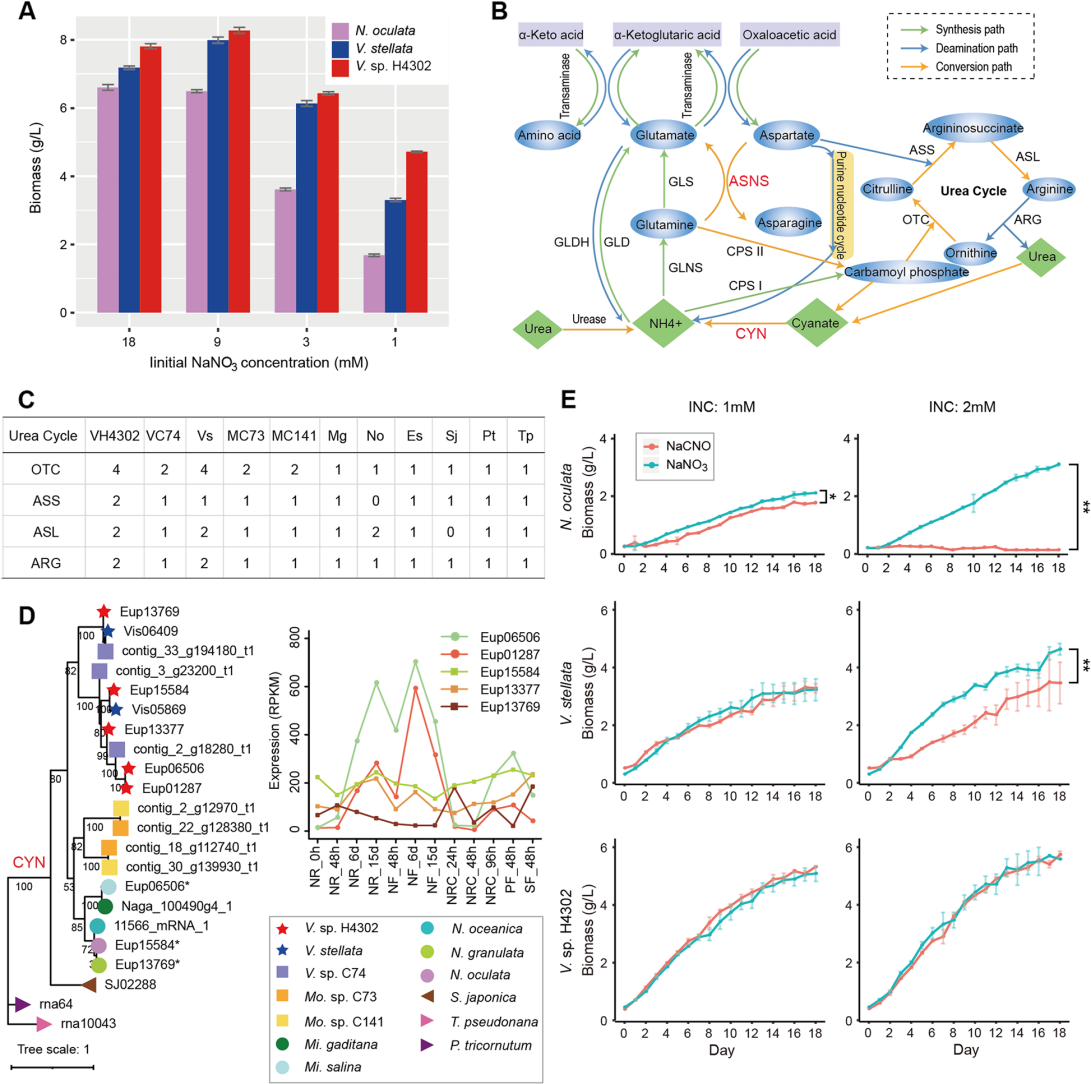
Evolution of *CYN* genes and tolerance to cyanate. **A** The histogram shows the biomass yield of three microalgae (*N. oculata*, *V. stellata*, and *V.* cf. *polyphem*) under four different initial NaNO_3_ concentrations. These data were adopted from Wang et al. [17]. **B** Overview of nitrogen metabolism pathways during amino acid synthesis and degradation. **C** The numbers of urea cycle genes in the two sequenced microalgae and nine other heterokontophytes. Abbreviations: VH4302, *V.* sp. H4302; VC74, *V.* sp. C74; Vs, *V. stellata*; MC73, *Mo*. sp. C73; MC141, *Mo*. sp. C141, Mg, *Mi. gaditana*; No, *N. oceanica*; Es, *E. siliculosus*; Sj, *S. japonica*; Pt, *P. tricornutum*; Tp, *T. pseudonana*. Genes: OTC, ornithine transcarbamylase; ASS: argininosuccinate synthase; ASL, argininosuccinate lyase; ARG, arginase. **D** Phylogenetic tree of *CYN* genes from thirteen heterokontophytes (left panel) and the RNA expression of five *CYN* genes of *V.* cf. *polyphem’*s (left panel). “*” means this gene is missing in released geneset and is predicted through a homology-based prediction in this study and the target gene ID is shown as above. **E** The biomass accumulation curves of *N. oculata*, *V. stellata*, and *V.* cf. *polyphem* with two kinds of nitrogen source (NaCNO and NaNO_3_) under two initial nitrogen concentrations (1 and 2 mM). Each data point represents the mean ± SD of three biological and technical triplicates. *, *P*-value < 0.05, **, *P*-value < 0.01

Nitrogen plays a pivotal role in the synthesis of both protein and nucleic acid. All the oleaginous microalgae cannot fix atmospheric N_2_ and rely directly on exogenous nitrogen. When nutrients in medium have been consumed in the late culture stage, the recycling and redistribution of nutrients in microalgal cells become very important. Nitrogen deprivation is one of the most effective stresses that induces lipid accumulation and affects biomass production in most oleaginous microalgae [42, 43], implying that nitrogen is a promising regulator of balancing biomass production and lipid content in oleaginous microalgae. In our previous study, *N. oculata*, *V. stellata*, and *V.* sp. H4302 were cultured in mBG-11 medium containing four different INCs, namely, 18, 9, 3, and 1 mM [19]. After 15 days of cultivation in all four INC conditions, *V*. sp. H4302 got the highest biomass concentration, followed by *V. stellata* and *N. oculata*. From 18 to 1 mM INC culture, *N*. *oculata* obtained a remarkable lower biomass concentration than the two microalgae of *Vischeria* at 3 mM INC, and *V. stellata* had significantly lower biomass than *V.* sp. H4302 at 1 mM INC (Fig. 5A), indicating that the growth of *V. stellata* and *V.* sp. H4302 was more tolerant to nitrogen-limiting stress, and *V.* sp. H4302 showed further tolerance.

In order to explore the molecular mechanism underlying the improved biomass yield of the two sequenced microalgae, especially *V.* sp. H4302, i under nitrogen-limiting conditions, we focused on genes related to nitrogen metabolism and amino acid metabolic pathways. The urea cycle plays an important function in deamination process of animals and it has been reported that all genes involved in the urea cycle could be found in *T. pseudonana* [44]. Here, we found that a complete set of urea cycle genes could be found in the two sequenced algae and most of the compared heterokontophytes in this study (Fig. 5C), suggesting that the urea cycle existed in the ancestor of Heterokontophyta. The expression of these genes could also be detected by transcriptome sequencing (Additional file 1: Fig. S10), meaning that the urea cycle was active in *V.* sp. H4302*.* In the “alanine, aspartate, and glutamate metabolism” pathway, we found there were seven and fifteen asparagine synthetase (*ASNS*) genes in *V.* sp. C74 and *V.* sp. H4302 respectively, compared to no more than four members in other algae compared of this paper (Additional file 1: Fig. S11A). Further, three and nine *ASNS* genes of the two *Vischeria* algae clustered into an isolated clade (Additional file 1: Fig. S11A), indicating sequence divergence and possible neo-/subfunctionalization evolution after the expansion and extra expansion in *V.* sp. H4302 afterdifferentiation of the two *Vischeria* algae. *ASNS* genes encode enzymes that catalyze the “ATP + H_2_O + L-aspartate + L-glutamine → AMP + diphosphate + H^+^  + L-asparagine + L-glutamate” reaction. In the amino acid degradation process, L-glutamate and L-aspartate are the two most widely known amino carriers. Most amino acids cannot be directly deaminated. The amino groups in these amino acids must first be transferred into ketoglutaric acid to generate L-glutamate and then be removed through L-glutamate oxidative deamination. Besides, L-glutamate’s amino group can be transferred to oxaloacetic acid to form L-aspartate which could deaminate through the purine nucleotide cycle or the urea cycle [45, 46] (Fig. 5B). In synthesis process, L-glutamate, L-glutamine, and carbamoyl phosphate are the only three bridge molecules between inorganic and organic ammonia [46]. L-glutamate provides the amino group for all other 18 amino acids directly or indirectly. L-glutamine also acts as nitrogen donor for three amino acids (tryptophan, histidine, and glutamine), as well as for purines and pyrimidines. In addition, L-glutamate and L-aspartate also act as carbon skeleton precursors for several amino acids [46]. It has been reported that overexpression of ASNS in *Arabidopsis* increased the tolerance of young seedlings when grown under nitrogen-limiting conditions [47]. ASNS helps to maintain the balance of these four important amino acids (L-aspartate, L-glutamate, L-asparagine, and L-glutamine), so the expanded *ASNS* genes in *V.* sp. H4302 may enhance its resistance under nitrogen-limiting conditions.

Another expanded gene in nitrogen metabolism is cyanate lyase (CYN) which can decompose cyanate to NH_3_ and CO_2_. Cyanate is a toxic compound, and it is also known as a regulator through modulating activities of other enzymes by its concentration levels [48–50]. It could be formed spontaneously during the degradation of carbamoyl phosphate and urea in microorganisms and microalgal cells [51, 52], and its concentrations increased in the late stationary phase of culture of two diatoms [53]. There was only one copy of the *CYN* gene in non-Eustigmatophyceae and Monodopsidaceae algae, while two, three, and five *CYN* copies were found in *V*. *stellata*, *V.* sp. C74, and *V.* sp. H4302, respectively. The two *Monodopsis* algae also had two *CYN* copies, while the topological structure of phylogenetic tree supports that the expansion in two genera were independent (Fig. 5D). Furthermore, two *CYN* copies (Eup06506 and Eup01287) from *V.* sp. H4302 that cluster away from the ancestor clade (containing *V*. *stellata* genes) showed a different expression pattern which had higher expression levels than other copies in late culture stage under nitrogen-free culture conditions, implying that this lineage-specific expansion with more sequence divergence in *V.* sp. H4302 may contribute more to its detoxification ability, especially in late culture or nitrogen limitation conditions that had high amino acid degradation activity or high nitrogen recycle demand. To test the tolerance of *N. oculata*, *V. stellata*, and *V.* sp. H4302 to cyanate and to determine their genetic capacity of utilizing and metabolizing cyanate, they were cultured in mBG-11 medium with sodium cyanate (NaCNO) or sodium nitrate (NaNO_3_) as sole nitrogen source, respectively*.* The results showed that the biomass concentrations of *V*. sp. H4302 or *V*. *stellata* did not differ significantly between the aforementioned two nitrogen sources under 1 mM INC condition, while the biomass concentration was significantly lower in NaCNO than in NaNO_3_ cultivation for *N. oculata* (Mann–Whitney *U* test, *P* < 0.05) (Fig. 5E, Additional file 2: Table S12). In cultivation with 2 mM INC condition, both of *V. stellata* and *N. oculata* had a significantly lower (*P* < 0.001) biomass concentration in NaCNO cultivation, especially for *N. oculata*, which eventually decreased in biomass and even died. However, the growth characteristics of *V*. sp. H4302 were still almost the same between these two nitrogen sources. These results significantly illustrated that the tolerance ability to cyanate was *V*. sp. H4302 > *V. stellata* > *N. oculata.*

As a complement to positive feedback, negative feedback is another common regulatory strategy. It seems like that cyanate acts as a negative regulator through reducing activities of enzymes and *CYN* genes act as the hedged regulator. The expansion of *CYN* genes can enhance the ability of cells to utilize cyanate for maintaining intracellular nitrogen balance under nitrogen limitation conditions. In the meantime, the expansion of *ASNS* genes in *V.* sp. H4302 may be another important auxiliary mechanism to maintain an active status under unbalanced nitrogen supply through enhancing the regulation ability of four important amino acids which act as containers and transfer stations both in amino acid synthesis and degradation process.

### Increasing of electron carriers in photosynthesis process for enhancing efficiency

**Fig. 6:**
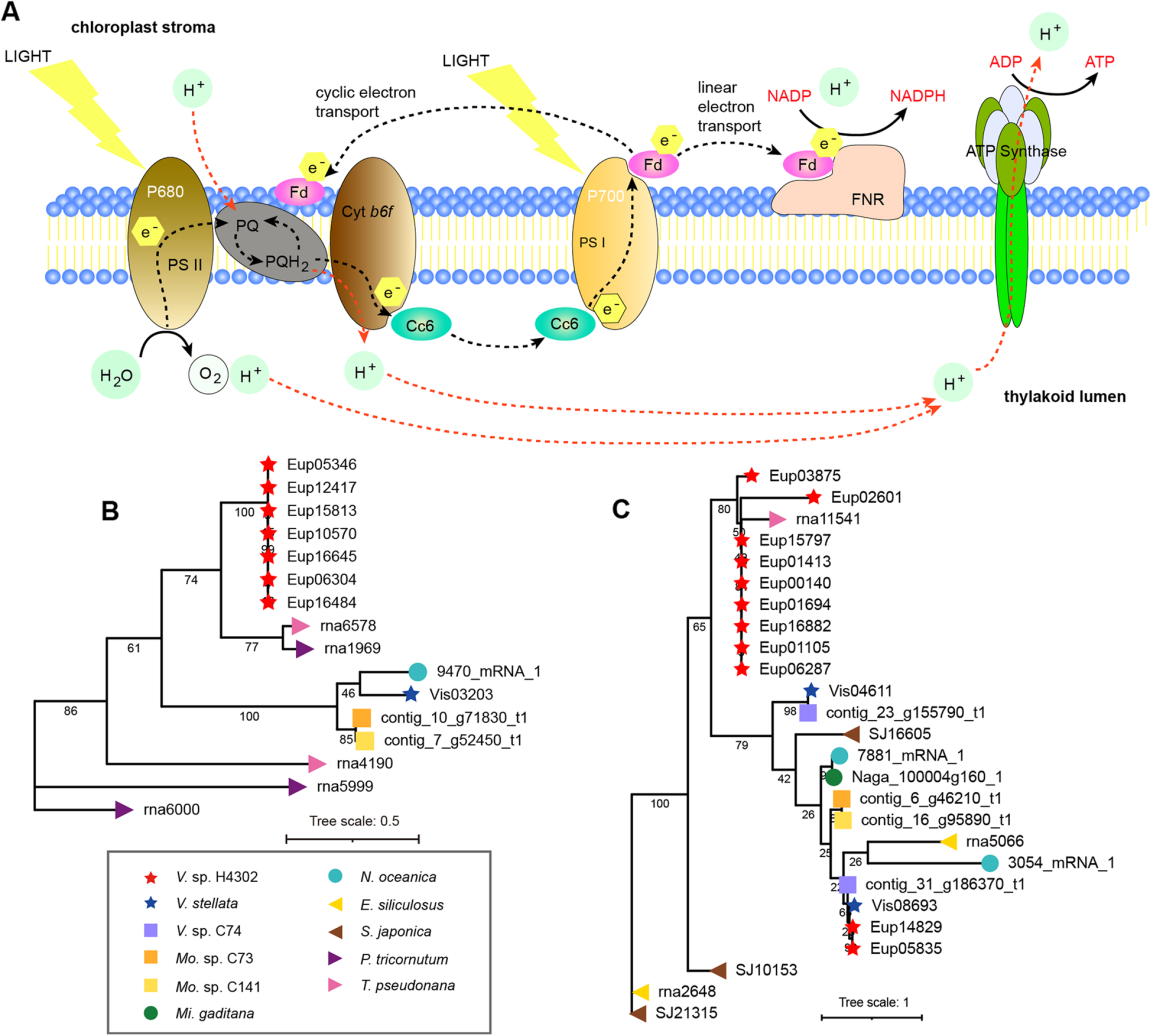
Phylogenetic analysis of two electron carrier genes. **A** Schematic overview of the electron transfer and the generation of ATP and NADPH in photosynthesis. **B** Phylogenetic trees of *Cc6* in eleven heterokontophytes. **C** Phylogenetic trees of *Fd* genes in eleven heterokontophytes

Photosynthesis is the ultimate physiological process to deliver sunlight as an energy source and converts water, carbon dioxide, and inorganic nutrients into biomass (including lipids) for obligate photoautotrophs. The basic mechanisms of the photoreaction stage include the following main modules: light harvest, electron transfer, ATP synthesis, and reducing power (NADPH) synthesis [54] (Fig. 6A). Interestingly, we found that the genes of two important electron carriers were expanded in *V.* sp. H4302*.* The electron carrier between the membrane-embedded cytochrome *b*6*f* complex and photosystem I (PSI) is plastocyanin (PC) in higher plants, or cytochrome *c*6 (Cc6) in some algae, or both in some other algae [55, 56]. The results showed that only *Cc6* was found in *V*. sp. H4302 and *V*. *stellata*, and *PC* was found in none of the nine background heterokontophytes, indicating that Cc6 acts as the sole electron carrier to PSI in heterokontophytes. There were seven copies of *Cc6* in *V.* sp. H4302, compared to one copy in other Eustigmatophyceae algae (Fig. 6B). In addition, the gene family encoding ferredoxin (Fd) was also expanded in *V.* sp. H4302 (Fig. 6C). Fd is one of the strongest soluble reductants ever found in cells, and it transfers electrons from the stromal side of PSI to ferredoxin NADP^+^ reductase (FNR) to produce NADPH [57]. The expansion of the two electron carriers may improve the electron transfer efficiency and further promote the photosynthetic efficiency in *V.* sp. H4302. Meanwhile, Fd also acts as a bottleneck and hub to distribute electrons to other metabolic pathways in the chloroplast, including transferring electrons to nitrite reductase, glutamate synthase, and thioredoxin reductase [58].

## Conclusions

Due to high content of lipid and excellent biomass yield, especially under nitrogen-limiting conditions, *V.* sp. H4302 and *V. stellata* have huge potential to promote the study of microalgae-based renewable biofuels. The high palmitoleic acid content in these two microalgae also indicates their potential value in the production of nutraceuticals for human health. Two high-quality genome assemblies presented here have enabled us to explore the evolutionary and molecular mechanisms associated with their special biological characteristics. Firstly, we found a battery of candidate genes that may be associated with the powerful lipid accumulation ability (lipid content and biomass yield) in *V.* sp. H4302 and *V. stellata* at multiple perspectives (FA and TAG synthesis, storage polysaccharides synthesis and hydrolysis, nitrogen metabolism, and photosynthesis efficiency). The most prominent gene is *CYN*, whose expansion enhanced the detoxification ability of the intermediate metabolite cyanate. Both lipid and sugar metabolism could influence the carbon flux distribution, but have little benefit on biomass yield. The evolutionary changes related to nitrogen metabolism might have conferred the capability for higher biomass yield under nitrogen-limiting conditions through maintaining better or longer photosynthesis working status in microalgal cells, and therefore produce more organic matter. This may also contribute to higher lipid content, as more carbon could be used for lipid synthesis. Secondly and unexpectedly, a WGD event was discovered in *V.* sp. H4302, which makes it an excellent candidate for the study of genome evolution following WGD events in microalgae. In summary, we provide two oleaginous microalgal genomes that are valuable resources to study TAG synthesis in microalgae, and we highlight several genomic changes that may account for their high TAG content and high biomass yield.

## Methods

### Genome sequencing and assembly

The genomes of *V.* sp. H4302 and *V. stellata* were sequenced both using the long-read sequencing technology and high-throughput chromosome conformation capture (Hi-C) technology. For long-read sequencing, one PacBio DNA library with an insert size of 20 kb was constructed following the protocol of the PacBio template preparation kit for two microalgae respectively. The *V.* sp. H4302 library was sequenced by a PacBio RSII instrument, and the *V. stellata* library was sequenced by a PacBio Sequel I instrument. For Hi-C sequencing, the Hi-C libraries were created as described previously [59]. In brief, cells were fixed with 2% formaldehyde. The cross-linked DNA was digested with MboI and the sticky ends were biotinylated by incubating with biotin-14-dATP and Klenow enzyme. After DNA purification and removal of biotin from unligated ends, Hi-C products were enriched and physically sheared to fragment sizes of 300–400 bp. The biotin-tagged Hi-C DNA was pulled down and processed into paired-end sequencing libraries, which were sequenced on the MGI2000 platform with pair-end (PE) 150 bp sequencing. For *V.* sp. H4302, one short insert size sequencing library (250 bp) was constructed according to the manufacturer’s instructions (Illumina) and was sequenced using Illumina Hiseq 2000 platform with read type of paired end 150 bp.

The genome assembly was executed through two steps, contig assembly using PacBio sequencing reads and chromosome assembly using Hi-C data. For contig assembly, raw PacBio sequencing reads were filtered firstly by Pacific Biosciences SMRT analysis software (v2.3.1) and the high-quality reads were corrected secondly by Falcon [60] (v0.2.2). Thirdly, the corrected reads of *V.* sp. H4302 were assembled to contigs by Falcon, and the reads of *V. stellata* were assembled by Smartdenovo (v1.0, https://github.com/ruanjue/smartdenovo) software, respectively. Fourthly, we corrected the raw contig assembly using Arrow from SMRT Link software (v7.0) for two assemblies with help of corrected PacBio reads. For assembly of *V.* sp. H4302, an extra correction was conducted using Pilon [61] (v1.22) software with the Illumina short reads. For chromosome assembly, it was constructed using Juicer + 3d_dna pipeline [62] with slight modifications. Briefly, Hi-C data were aligned to the contig assembly firstly using Bowtie2 [63] (v2.2.5) software. Then Juicer [64] and 3d_dna [62] were used for assembly clustering, ordering, orienting, and evaluating. The Juicerbox [65] (v1.11.08) was used for manual corrections. The completeness of assembly was assessed using Benchmarking Universal Single-Copy Orthologs (BUSCO) pipeline [21] with orthologs database of stramenopiles_odb10.

### SNP calling

The high-quality Illumina reads were aligned to assembly of *V.* sp. H4302 using BWA [66] (v2.0) software. The alignment result was sorted and duplications were removed using Samtools [67] (v1.9). The variations were called using HaplotypeCaller program from Genome Analysis Toolkit [68] (GATK, v4.1.2.0), and the SNPs were selected using SelectVariants program from GATK. Finally, we filtered the SNPs using VariantFiltration from GATK with parameters “–filter QD < 2.0 –filter MQ < 40.0.”

### Transcriptome sequencing

We constructed 12 cDNA libraries using the Illumina TruSeq RNA sample preparation kit according to the manufacturer’s instructions, including four nitrogen repletion (NR, INC 18 mM) cultures (sampling time: 0 h, 48 h, 6 days, and 15 days); three nitrogen-free (NF, INC 0 mM) cultures (sampling time: 48 h, 6 days, and 15 days); three nitrogen recovery cultures (NRC, 15 days of NF culture, followed by transfer into 18 mM of INC medium; sampling time: 48 h, 6 days, and 15 days); a phosphorus-free (PF) culture (sampling time: 48 h); and a sulfur-free (SF) culture (sampling time: 48 h) (Additional file 1: Fig. S12). The sequencing was performed with an Illumina Hiseq 2000 platform with the PE150 strategy, yielding more than 29 million reads for each library (Additional file 1: Table S11). RNA reads were mapped to the genome with Hisat2 [69] (v2.1.0). Stringtie [70] (v1.0.4) was used to assemble transcripts in each sample and merge them into combined transcripts. We quantitated gene expression using unique mapping reads. One read was quantitated to one gene when its mapping region had more than 50% overlap with exons. We normalized the expression using the reads per kilobase of transcript per million mapped reads (RPKM).

### Repetitive element prediction

We predicted the repetitive elements using a combination of homology-based and de novo approaches. For homology-based repeats, RepeatMasker and RepeatProteinMask (http://www.repeatmasker.org/, v3.3.0) were employed to identify repetitive elements based on homologous search against libraries of Repbase (release 20.04) using the parameters “-nolow -no_is -norna -parallel 1” and “-noLowSimple –pvalue 1e-4,” respectively. De novo repeat prediction was conducted in three steps. First, the ab initio prediction program Piler [71] (v1.0), Repeatscout [72] (v1.0.5), and LTRharvest [73] (v1.5.9) were employed to build the de novo repeat library. Second, putative protein-coding genes were removed from the library by alignment to the Swiss-Prot database. Third, de novo repeats from the three predictions were merged and RepeatMasker was used again to find repeats in the genome against this de novo library. We also performed RE re-predictions for twelve compared algae using this pipeline to reduce system errors from different software.

### Gene prediction and functional annotation

Evidence from homolog-based and RNA-seq data were employed to predict gene models of *V.* sp. H4302. Homolog-based prediction was performed using a TBLASTN + Genewise [74] pipeline through aligning protein sequences of nine species (Additional file 1: Table S8) to the assembly. Firstly, the protein sequences of each species were aligned to the assembly using TBLASTN with an e-value threshold of < 1e − 5, then the high-scoring pairs (HSPs) were conjoined using Solar (https://github.com/gigascience/papers/tree/master/zhou2013/MT_annotation_BGI/solar) to determine the rough genomic region for each gene. Thirdly, the conjoined regions were extracted from the genome, with a 2-kb extension both upstream and downstream, and aligned again with the protein sequences to define gene models using Genewise [74] (v2.4.1). We merged the results of different species and removed redundancy based on the score of Genewise. We also filtered the gene models with less than 30% coverage and with more than 50% overlap with repetitive element regions. For RNA evidence, we used RNA transcripts to extend the gene models from the Genewise pipeline to predict open reading frames (ORFs). The extension strategy refers to a published Ensembl Gene Annotation System [75] (Fig. 3 of the reference paper). Besides, we predicted some novel genes that were not predicted by the Genewise pipeline. We trained a fifth-order Markov model using intact gene models from the Genewise pipeline and used this model to predict ORFs for RNA transcripts. The ORFs that did not overlap with the Genewise geneset were added to generate the final geneset of *V.* sp. H4302. Since RNA-seq data for *V. stellata* were not available and only homolog-based prediction was conducted, the gene models of *V.* sp. H4302 were also used to predict genes of *V. stellata*.

We annotated the function of predicted gene models by aligning the protein sequences to the Swiss-Prot [76] (release Jun 2019), NCBI Nr (release Sep 2017), and KEGG [23] (release 89) databases. The gene symbols and pathways were assigned based on the best blast hit against the Swiss-Prot and KEGG databases, respectively. GO terms, motifs, and domains of protein sequences were annotated using InterProScan [77] (release 5.3) by searching against publicly available databases, including Pfam, PRINTS, PANTHER, PROSITE, ProDom, and SMART.

### Gene cluster analysis

A hierarchical clustering analysis was performed using protein sequences of 14 species, including *V*. sp. H4302, *V*. *stellata*, and twelve species with previously published genomes [6–8, 22, 44, 78–80] (Additional file 1: Table S8). If a gene had more than one transcript, the longest transcript was used. Considering the low BUSCO assessment for genesets of *Microchloropsis salina* (former name: *Nannochloropsis salina*) CCMP53*7*, *Nannochloropsis oculata* CCMP525, and *Nannochloropsis granulata* CCMP529 (Additional file 1: Table S7), we performed a homology prediction for these three microalgae using *V.* sp. H4302’s geneset as reference. After data processing, BLASTp (blast-2.2.26) was employed to do an all-vs-all alignment based on protein sequences to identify the potential homologous sequences with e-value < 1e − 5. The blast results were clustered into gene families using OrthoMCL [81] with default parameters. Based on this cluster result, we identified lineage-specific genes in species or clades. We performed KEGG functional enrichment analysis for lineage-specific genes using a hypergeometric test using PHYPER in R and tested the false discovery rate using QVALUE in R.

### Whole-genome duplication (WGD) analysis

We analyzed WGD events using WGD software [82] which was based on synonymous substitution rate (Ks) distribution. The main steps included (1) using the mcl module to identify paralogous gene pairs within *V*. sp. H4302, *Vischeria* sp. C74, and *V*. *stellata* respectively; (2) using the dmd module to identify orthologous gene pairs between *V*. sp. H4302 and *V*. sp. C74, and *V*. sp. H4302 and *V*. *stellata*; (3) using the ksd module to calculate Ks distribution for above five groups of gene pairs. Finally, we compared the Ks distribution of different groups from intra-species or inter-species to identify the WGD event. In addition, we used Blastp + MCscanX [83] pipeline to identify the collinearity of orthologous or paralogous gene pairs. Firstly, an all-vs-all Blastp alignment was done using protein sequences. Then the MCscanX [83] was used to detect homologous blocks intra-species or inter-species respectively. Syntenic orthologous blocks between *V*. sp. H4302 and *V*. *stellata* were visualized using NGenomeSyn (https://github.com/hewm2008/NGenomeSyn/) software. The syntenic paralogous blocks within *V*. sp. H4302 were visualized using Circos [84] software. The Ks of the syntenic orthologous gene pairs was calculated using Kaks_Calculator [85] (version 2.0) with default parameter.

### Phylogenetics and divergence time

In total, 249 one-to-one orthologous genes among fourteen algae were obtained from the OrthoMCL cluster result. Besides, considering the WGD events in *V.* sp. H4302, we also identified 698 two-to-one orthologous genes (two in *V.* sp. H4302, one in other species) and selected the longer one for *V.* sp. H4302. Thus, we obtained 947 orthologous gene pairs. The protein sequences of each ortholog were aligned using MUSCLE [86] (v3.8.31) with default parameters. The poorly aligned regions were removed using trimAl [87] (v1.2) with the parameter “-gt 0.8 -st 0.01.” Then we converted the data into nucleotide alignment by tracing the coding relationship and extracted the phase 0 and phase 1 sites. We linked all alignments to form a concatenated alignment. Finally, we used RAxML [88] (v8.2) to construct phylogenetic trees using the GTRGAMMA model. We estimated divergence times using PAML MCMCTREE [89] (v4.5). The Markov chain Monte Carlo (MCMC) process was run for 200,000 iterations with a sample frequency of 500 after a burn-in of 20,000 iterations. The following constraints were used for time calibrations: (i) the divergence time between *E. silliculosus* and *S. japonica* is 127–218 mya [90, 91]; (ii) the divergence time between *T. pseudonana* and *P. tricornutum* is 201–221 mya [92]; (iii) the divergence time between *P. tricornutum* and *S. japonica* is 438–819 mya (http://www.timetree.org/).

### Gene family analysis

The gene cluster analysis from the OrthoMCL pipeline revealed some candidate genes for which the copy number changed or the sequence evolved in a divergent manner from the background species. To further investigate the evolution of candidate genes, we first identified the whole gene families based on motif annotation or Swiss-Prot annotation. Then we constructed a phylogenetic tree for each gene family. We aligned the protein sequences of each gene family using MUSCLE and filtered the alignments using trimAl with the parameter “-gt 0.8.” The phylogenetic tree was constructed using IQ-TREE [93] (v1.6.6) with parameters “-b 100 -m MFP”. In this study, we constructed the phylogenetic tree for nine gene families, including *DGAT* (Pfam motif: PF03982), *PDAT* (Pfam motif: PF02450), *FAD9* (InterProScan motif: IPR015876 and IPR005067), *β-1,3-glucan synthase* (Pfam motif: PF02364), *β-1,3-glucanase* (Pfam motif: PF00722), *ASNS* (Pfam motif: PF00733), *CYN* (InterPro motif: IPR008076), *Cc6* (Pfam motif: PF13442), and *Fd* (best hit to petF genes in Swiss-Prot). The gene IDs of all mentioned genes in the manuscript are provided in Additional file 3: Table S13.

### Algae cultivation

In order to test the tolerance ability to cyanate, we cultured *V.* sp. H4302, *V*. *stellata*, and *N. oculata* in mBG-11 media with 1 mM or 2 mM initial nitrogen concentrations of sodium cyanate (NaCNO) and sodium nitrate (NaNO_3_) as sole nitrogen resource, respectively. Other shared culture conditions including cultivation in bubbling glass column photobioreactors (Ø6 cm × 60 cm), continuous illumination of 200 μmol/(m^2^·s) provided by fluorescent light, and bubbled by compressed air enriched with 1% CO_2_ (v/v). Biomass concentration was measured as follows: 5 mL of algal cultures were collected every 3 days or at the end of cultivation and filtered through a pre-weighed 0.45 μm GF/B membrane (M0). The membrane containing algal cells was dried in an oven overnight at 105 °C and weighed as M1. The biomass dry weight (DW, g/L) was then determined as the difference between M0 and M1 and calculated as (M1 − M0) × 200.

## Supplementary Information

**Additional file 1: Figs. S1 and S2.** Hi-C heatmap of two assemblies. **Figs. S3 and S4.** Paralogous blocks and gene pairs within genome of *V.* sp. H4302. **Fig. S5.** Evolution and expression bias of paralogous gene pairs in *V.* sp. H4302. **Fig. S6.** Overview of chloroplastic fatty acid synthesis pathway and gene number. **Figs. S7, S8 and S9**, and **S11.** Phylogenetic analysis of *FAD9, β-1,3-glucan synthase, β-glucanase*, and *ASNS* genes respectively. **Fig. S10.** RNA expression of urea cycle genes in *V.* sp. H4302. **Fig. S12.** Biomass accumulation curves of *V.* sp. H4302 under the different conditions. **Tables S1, S2, S3 and S4.** Statistics of sequencing data and two assemblies. **Tables S5 and S7.** BUSCO evaluation for assemblies and genesets. **Table S6.** Statistics of repetitive elements. **Table S8.** Key resources table. **Tables S9 and S10.** Significant enriched KEGG pathways for lineage-specific genes of Eustigmatophyceae and *V.* sp. H4302 respectively. **Table S11.** Statistics of transcriptome data.

**Additional file 2: Table S12.** Biomass of *V.* sp. H4302, *V. stellata*, and *N. oculata* in 18 days cultivations with 1 mM or 2 mM initial nitrogen concentrations of sodium cyanateand sodium nitrateas sole nitrogen resource, respectively.

**Additional file 3: Table S13.** The gene IDs of all mentioned genes in the manuscript.

## Data Availability

All data generated or analyzed during this study are included in this published article, its supplementary information files and publicly available repositories. The sequencing data and genome assemblies of *V.* sp. CAUP H4302 and *V*. *stellata* SAG 33.83 are deposited at CNGB database under accession number of CNP0000525 (https://db.cngb.org/search/?q=CNP0000525). The sequence data were also deposited in the Sequence Read Archive (https://www.ncbi.nlm.nih.gov/sra) under accession numbers PRJNA680677.

## Abbreviations

WGD: Whole-genome duplication
DW: Dry weight
INC: Initial nitrogen concentrations
POA: Palmitoleic acid
Hi-C: High-throughput chromosome conformation capture
REs: Repetitive elements
Ks: Synonymous substitution rate
Ka: Nonsynonymous substitution rate
TAG: Triacylglycerol
FAS: Fatty acid synthesis
GLS: Glycerolipid synthesis
FAD9: Delta-9 desaturase
DGAT: Acyl-CoA:diacylglcerol acyltransferase
PDAT: Phospholipid:diacylglcerol acyltransferase
NR: Nitrogen repletion
NF: Nitrogen-free
NRC: Nitrogen recovery
GS: β-1,3-Glucan synthase
ASNS: Asparagine synthetase
CYN: Cyanate lyase
Cc6: Cytochrome *c*6
Fd: Ferredoxin
