# Supplementary material for: The genomes of Vischeria oleaginous microalgae shed light on the molecular basis of hyper-accumulation of lipids
Source: BMC Biol. 2023 Jun 6;21:133. doi: 10.1186/s12915-023-01618-x (PMC10245428; doi:10.1186/s12915-023-01618-x)
Supplement: Supplementary file 1 — Additional file 1: Figs. S1 and S2. Hi-C heatmap of two assemblies. Figs. S3 and S4. Paralogous blocks and gene pairs within genome of V. sp. H4302. Fig. S5. Evolution and expression bias of paralogous gene pairs in V. sp. H4302. Fig. S6. Overview of chloroplastic fatty acid synthesis pathway and gene number. Figs. S7, S8 and S9, and S11. Phylogenetic analysis of FAD9, β-1,3-glucan synthase, β-glucanase, and ASNS genes respectively. Fig. S10. RNA expression of urea cycle genes in V. sp. H4302. Fig. S12. Biomass accumulation curves of V. sp. H4302 under the different conditions. Tables S1, S2, S3 and S4. Statistics of sequencing data and two assemblies. Tables S5 and S7. BUSCO evaluation for assemblies and genesets. Table S6. Statistics of repetitive elements. Table S8. Key resources table. Tables S9 and S10. Significant enriched KEGG pathways for lineage-specific genes of Eustigmatophyceae and V. sp. H4302 respectively. Table S11. Statistics of transcriptome data. [file 12915_2023_1618_MOESM1_ESM.docx]

**This PDF file includes:**

Figure S1 to S12

Tables S1 to S11

# Supplementary Figure


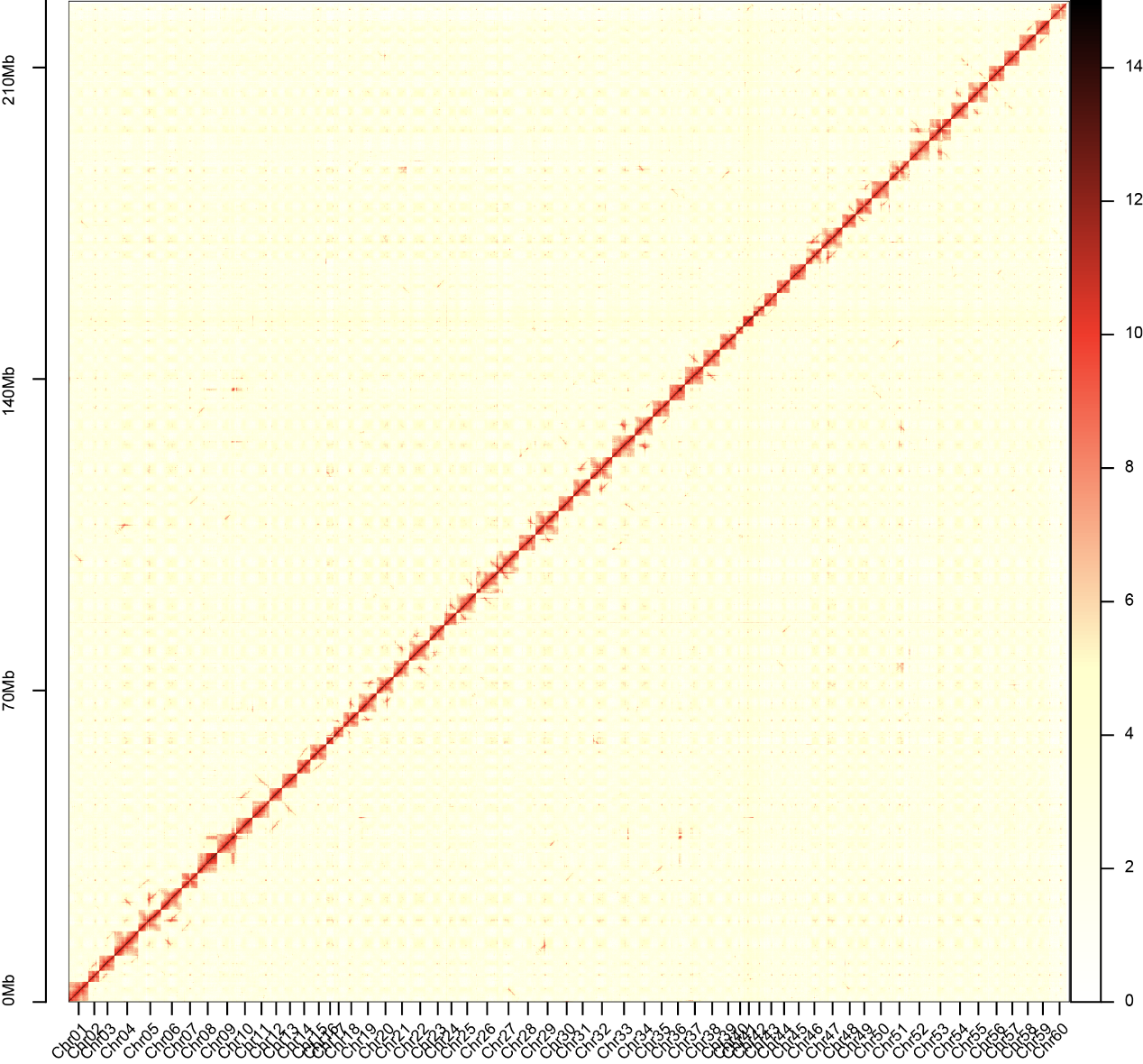


**Fig. S1** The genome-wide Hi-C heatmap of the *V.* sp. H4302 assembly.


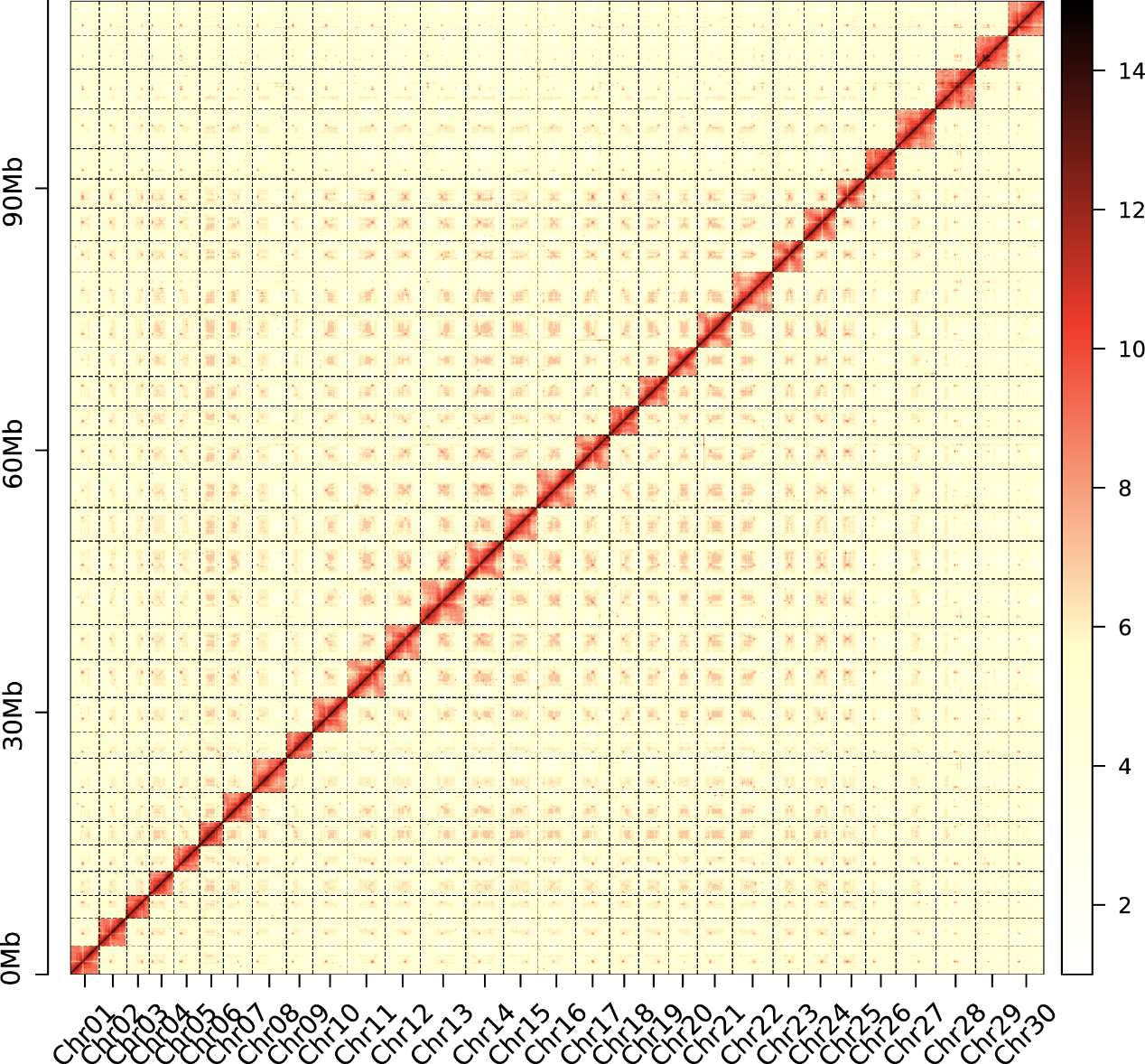


**Fig. S2** The genome-wide Hi-C heatmap of the *V. stellata* assembly.


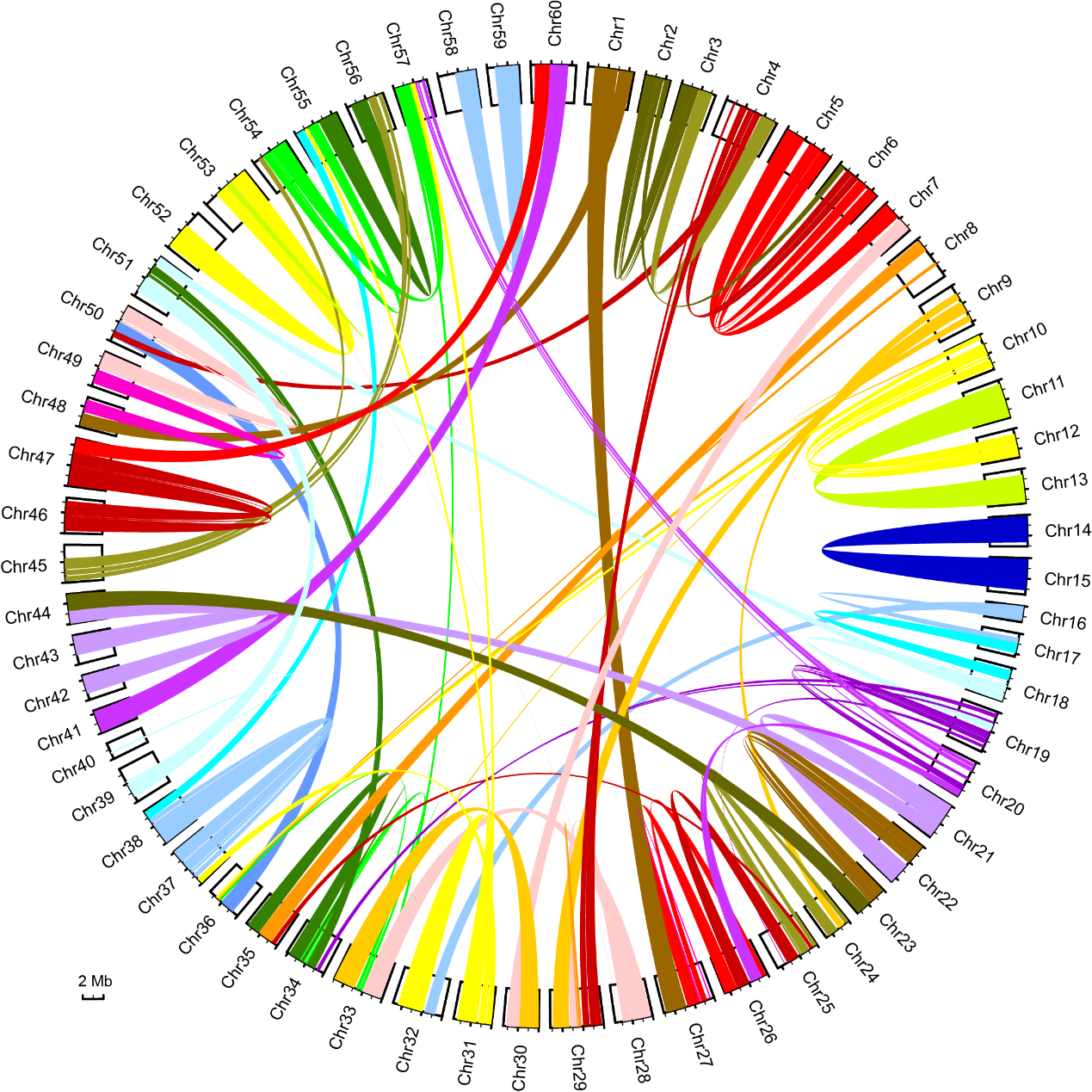


**Fig. S3** The collinearity paralogous blocks (≥ 5 gene pairs) within the genome of *V.* sp. H4302*.*


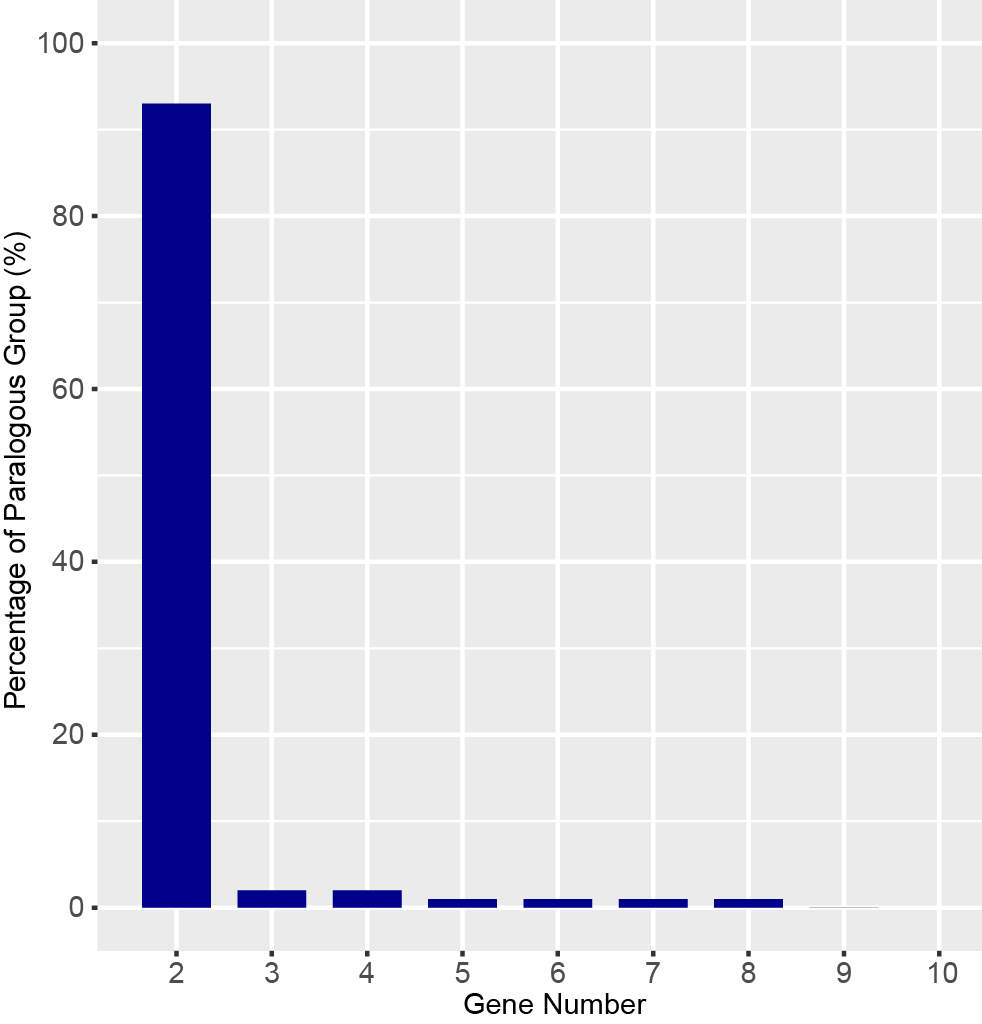


**Fig. S4** The gene number distribution of paralogous gene groups within *V.* sp. H4302.

**
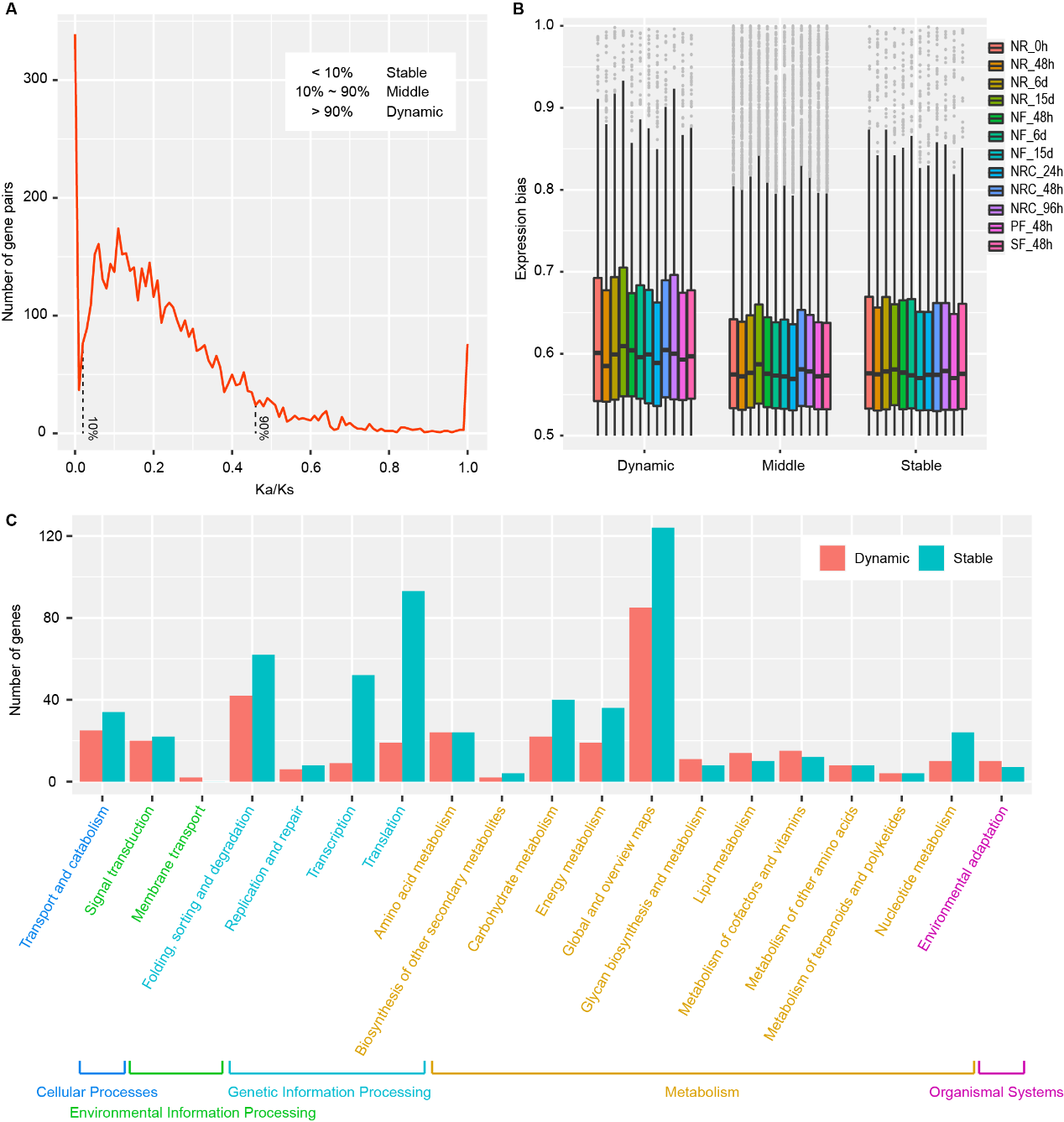
**

**Fig. S5** Evolution and expression bias of paralogous gene pairs in *V.* sp. H4302*.* (**A**) Distribution of Ka/Ks ratio of paralogous gene pairs. The 10% most stable and 10% most dynamic pairs were defined. (**B**) Box plots of paralog expression bias of each category (stable, middle, and dynamic) across 12 samples. Expression bias is calculated using the higher expression / the total expression of gene pairs. (**C**) KEGG annotation of the 10% most stable and 10% most dynamic paralogous gene pairs.


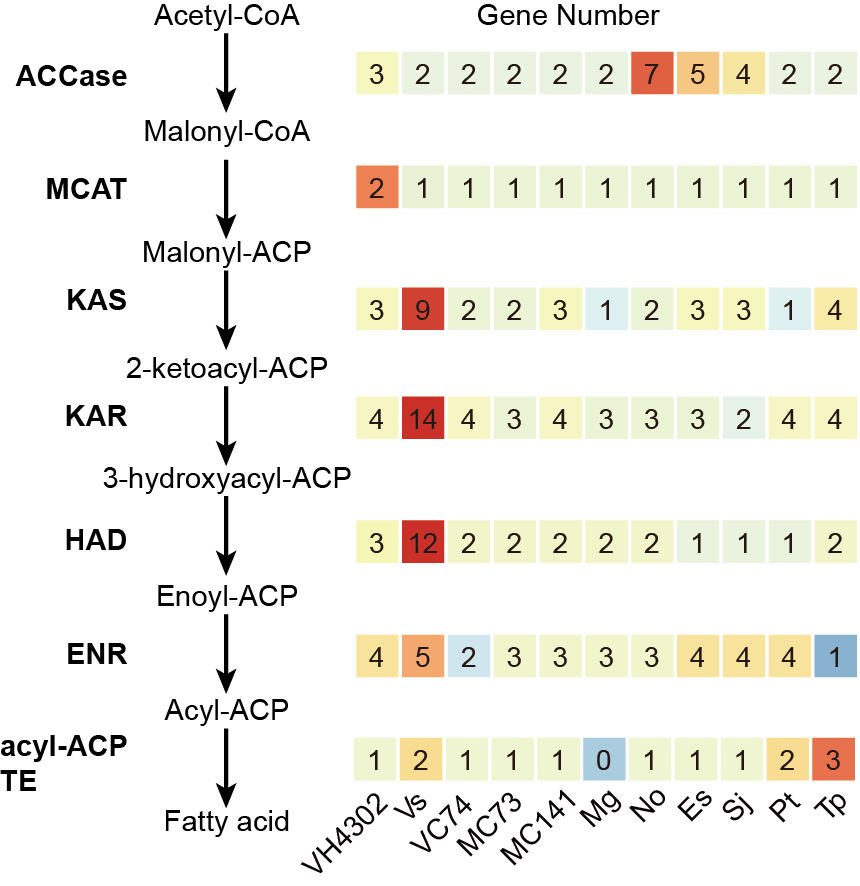


**Fig. S6** Chloroplastic fatty acid synthesis pathway [1] and gene number in eleven algae. The colour of rectangle is reflection of relative size of the gene number in each row and the gene number is shown in rectangle. Genes: ACCase, acetyl-CoA carboxylase; MCAT, Malonyl-CoA-acyl carrier protein transacylase; KAS, 3-oxoacyl-[acyl-carrier-protein] synthase (eliminating the mitochondrial members); KAR, 3-oxoacyl-[acyl-carrier-protein] reductase; HAD, hydroxyacyl-CoA dehydrogenase; ENR, Enoyl-[acyl-carrier-protein] reductase; acyl-ACP TE, Acyl-protein thioesterase. Species: VH4302, *V.* sp. H4302; Vs, *V. stellata*; VC74, *V.* sp. C74; MC73, *Mo.* sp. C73; MC141, *Mo.* sp. C141; Mg, *Mi. gaditana*; No, *N. oceanica*; Es, *E. siliculosus*; Sj, *S. japonica*; Pt, *P. tricornutum*; Tp, *T. pseudonana*.


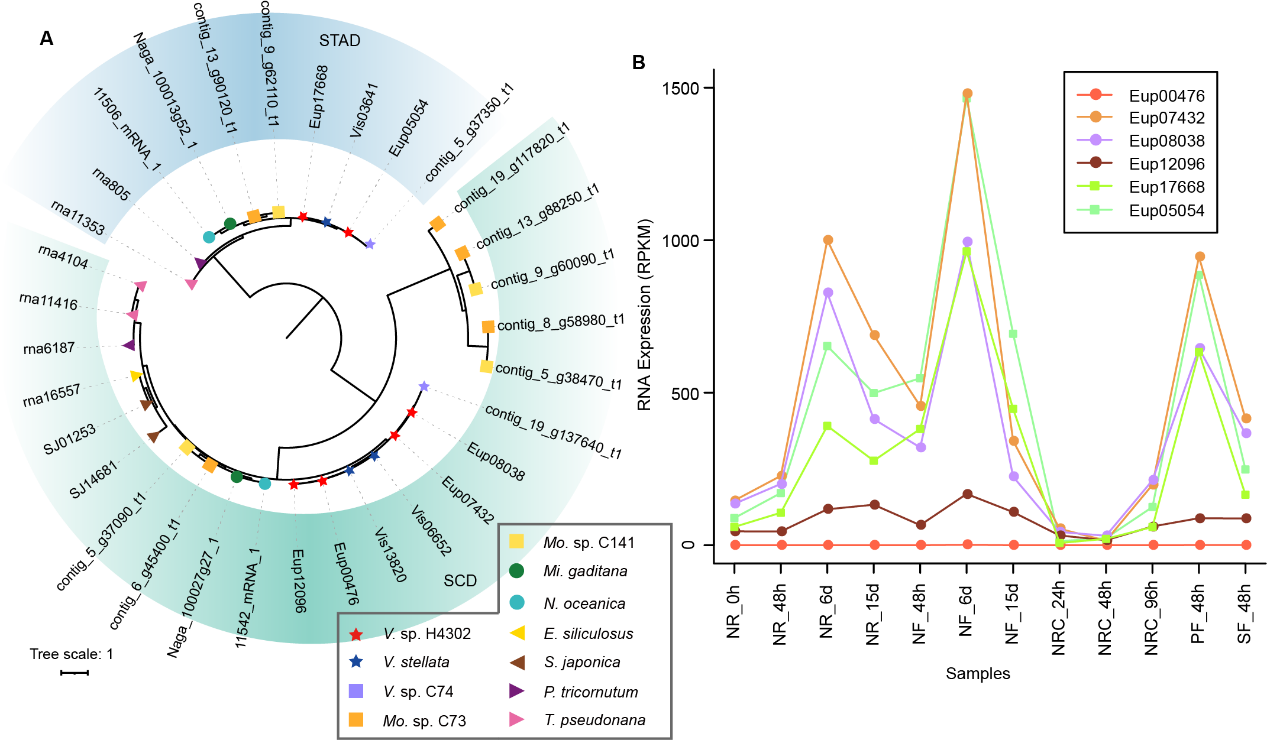


**Fig. S7** Phylogenetic analysis and expression of delta-9 desaturase (FAD9) genes. (**A**) The phylogenetic tree of FAD9 genes. SCD, stearoyl-CoA desaturase 5, InterProScan motif: IPR015876; STAD, stearoyl-[acyl-carrier-protein] 9-desaturase, InterProScan motif: IPR005067. (**B**) RNA expression of FAD9 genes in *V.* sp. H4302.


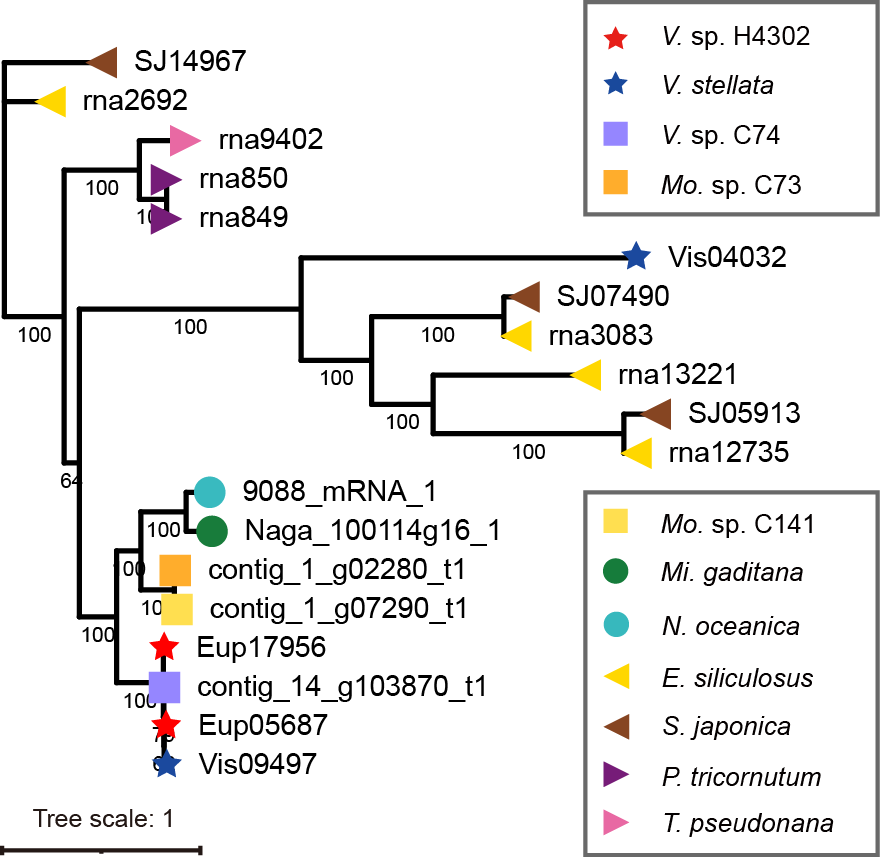


**Fig. S8** The phylogenetic tree of *β-1,3-glucan synthase* genes (Pfam motif: PF02364) in two assemblies and nine other heterokontophytes.


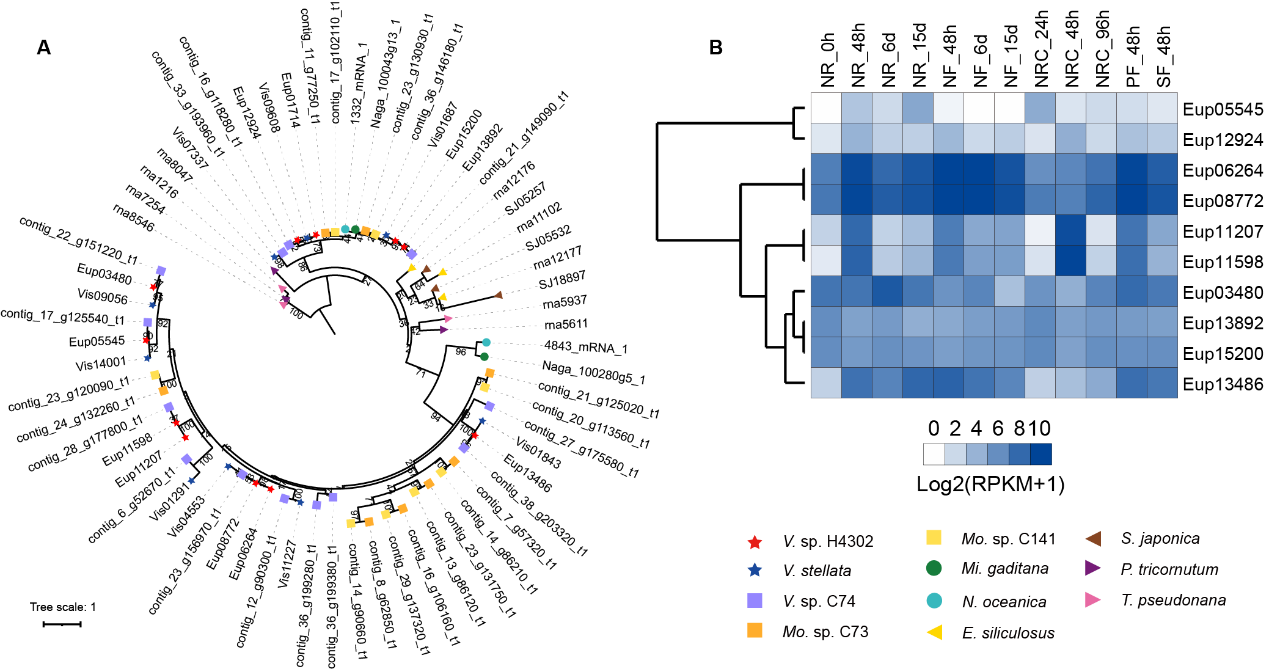


**Fig. S9** Phylogenetic analysis and expression of *β-glucanase*. (**A**) The phylogenetic tree of *β-glucanase* genes (Pfam motif: PF00722) in eleven heterokontophytes. (**B**) RNA expression of *β-glucanase* genes in *V.* sp. H4302*.*

**
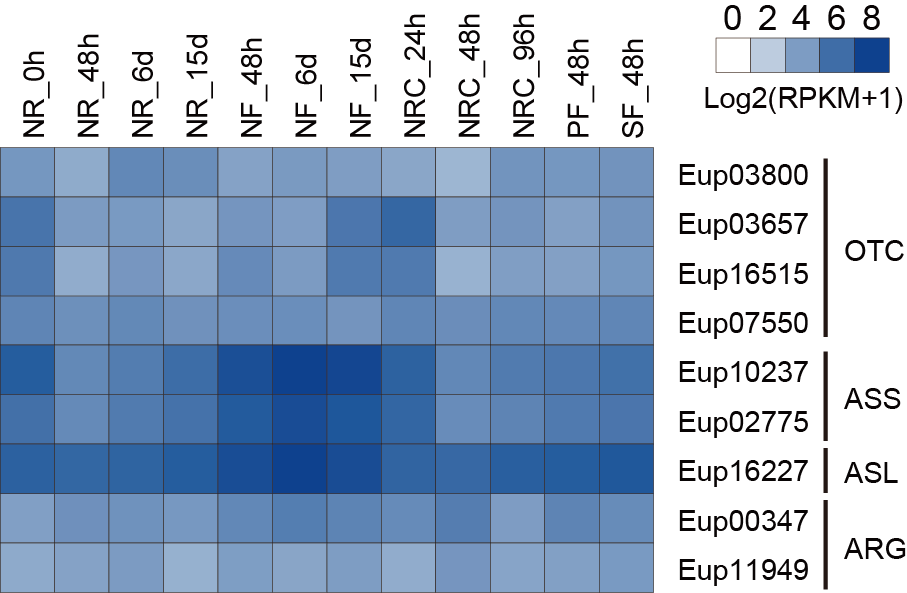
**

**Fig. S10** RNA expression of urea cycle genes in *V.* sp. H4302*.*


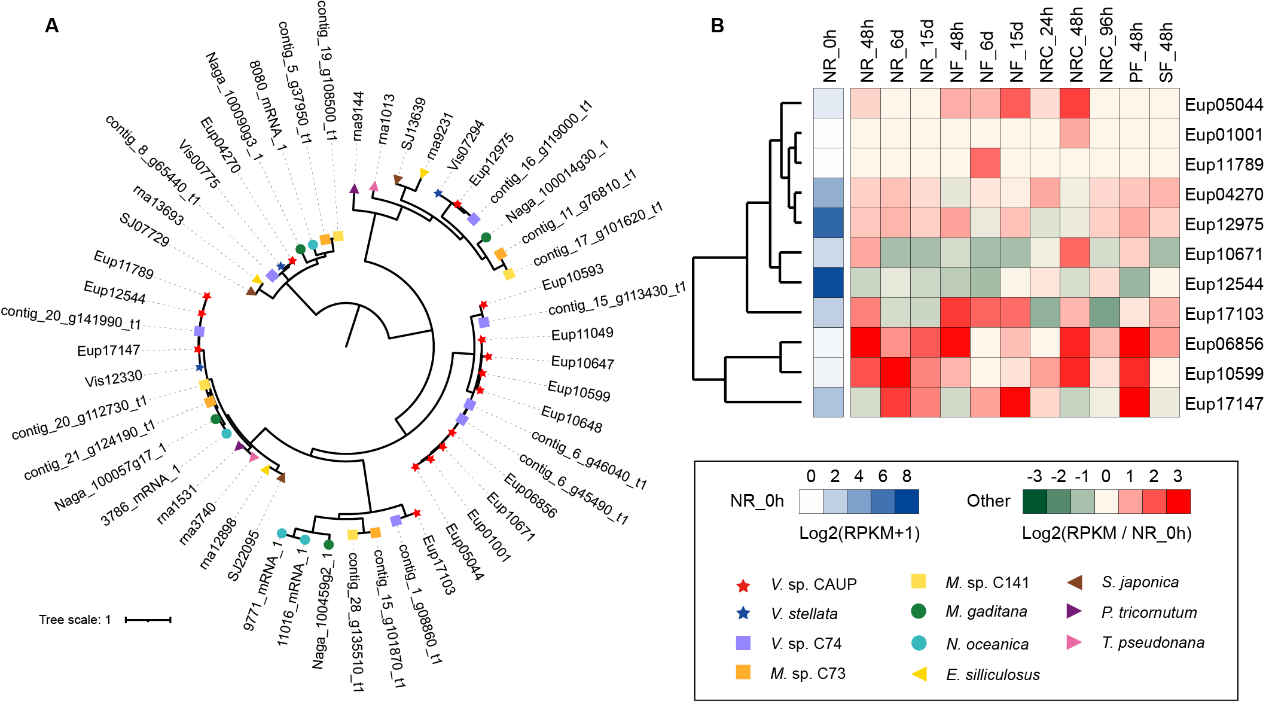


**Fig. S11** Phylogenetic analysis and expression of *ASNS* genes. (**A**) The phylogenetic tree of *ASNS* genes (Pfam motif: PF00733) in eleven heterokontophytes. (**B**) RNA expression of *ASNS* genes in *V.* sp. H4302.


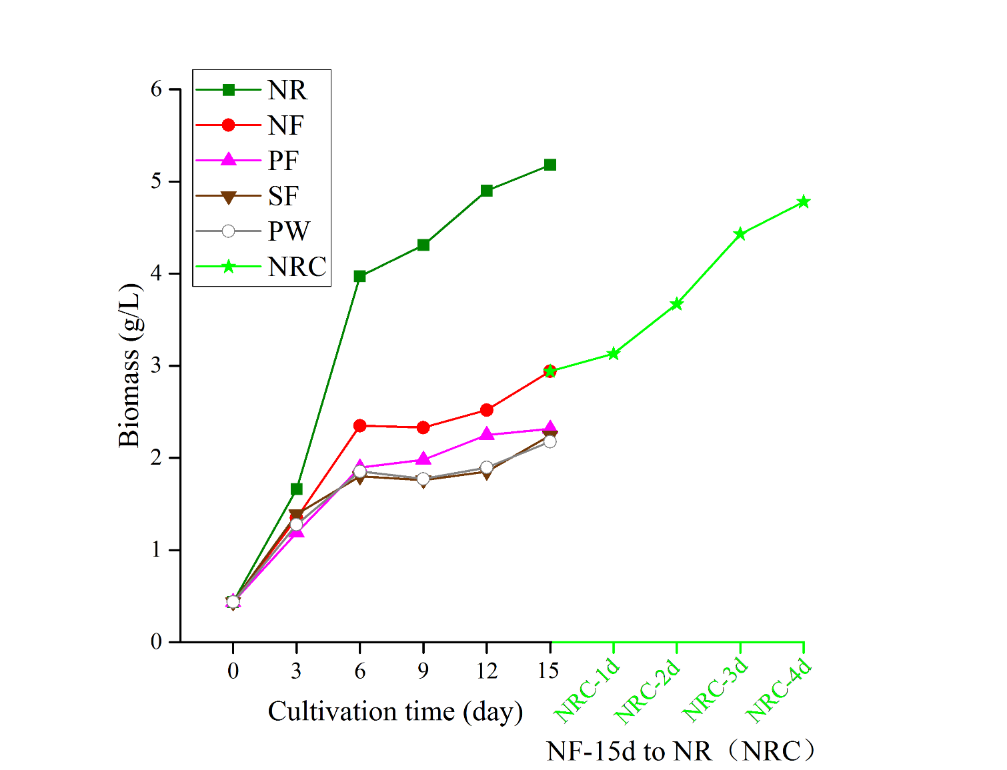


**Fig. S12** Biomass accumulation curves of *V.* sp. H4302 under the different conditions. Abbreviations: NR, nitrogen repletion, initial nitrogen concentrations (INC) 18 mM; NF, nitrogen free, INC 0 mM; NRC, nitrogen recovery, 15 days NF cultures were transferred into 18 mM of INC medium; PF, phosphorus free; SF, sulphur free; PW, pure water.

# Supplementary Tables

**Table S1** Statistics of long reads sequencing data

| Species | *V.* sp. H4302 | *V. stellata* |
| --- | --- | --- |
| Sequencer | PacBio RSII | PacBio Sequal I |
| Library Size | 20 kb | 20 kb |
| Mean Reads Length | 8 kb | 11 kb |
| Sequence Depth (X) | 69 | 136 |
| Total Data (Gb) | 19.5 | 16.6 |

**Table S2** Results of genome assembly for *V.* sp. H4302

| Assembly Statistics | Scaffold | Contig |
| --- | --- | --- |
| Total length（bp） | 229,764,204 | 229,454,704 |
| Sequence Number | 372 | 991 |
| Longest length(bp) | 5,392,295 | 2,976,631 |
| N10 | 5,000,162 | 1,546,140 |
| N20 | 4,580,603 | 1,278,558 |
| N30 | 4,458,527 | 1,035,504 |
| N40 | 4,149,828 | 823,644 |
| N50 | 3,753,770 | 680,399 |
| N60 | 3,671,774 | 461,771 |
| N70 | 3,545,702 | 371,315 |
| N80 | 3,359,193 | 261,712 |
| N90 | 2,828,487 | 153,675 |

**Table S3** Statistics of Hi-C sequencing data

| Species | Reads Number | Base Number | Reads Length | Q20 |
| --- | --- | --- | --- | --- |
| *V.* sp. H4302 | 394,137,250 | 59,120,587,500 | 150 | 97.72% |
| *V. stellata* | 314,997,950 | 47,249,692,500 | 150 | 97.72% |

**Table S4** Results of genome assembly for *V. stellata*

| Assembly Statistics | Scaffold | Contig |
| --- | --- | --- |
| Total length（bp） | 115,377,152 | 115,281,652 |
| Sequence Number | 100 | 291 |
| Longest length (bp) | 5,188,432 | 3,633,963 |
| N10 | 4,566,250 | 2,614,066 |
| N20 | 4,362,016 | 2,150,401 |
| N30 | 4,032,181 | 1,698,141 |
| N40 | 4,013,335 | 1,280,793 |
| N50 | 3,848,697 | 1,088,283 |
| N60 | 3,569,920 | 815,517 |
| N70 | 3,327,934 | 569,274 |
| N80 | 3,283,641 | 341,691 |
| N90 | 2,774,522 | 186,738 |

**Table S5** BUSCO assessing of two assemblies and other closely related species genomes

| **Species** | **Genome** | | | |
| --- | --- | --- | --- | --- |
|  | **Complete** | **Duplicated** | **Fragmented** | **Missing** |
| *V.* sp. H4302 | 92% | 56% | 6% | 2% |
| *V. stellata* | 90% | 6% | 6% | 4% |
| *V.* sp. C74 | 92% | 0% | 6% | 2% |
| *Mo.* sp. C73 | 85% | 0% | 11% | 4% |
| *Mo.* sp. C141 | 89% | 0% | 6% | 5% |
| *Mi. gaditana* B-31 | 91% | 0% | 4% | 5% |
| *Mi. salina* CCMP53*7* | 82% | 0% | 10% | 8% |
| *N. oceanica* CCMP1779 | 84% | 2% | 6% | 10% |
| *N. ranula* CCMP525 | 59% | 0% | 16% | 25% |
| *N. ranulate* CCMP529 | 70% | 0% | 11% | 19% |

Note: orthologs database is stramenopiles_odb10.

**Table S6** Repetitive elements statistics in two assemblies and nine published heterokontophytes

| **Species** | | **Genome** | **DNA** | **LINE** | **SINE** | **LTR** | **SSR** | **Total** |
| --- | --- | --- | --- | --- | --- | --- | --- | --- |
| *V.* sp. H4302 | Length | 230,059,433 | 90,261,519 | 34,445,173 | 101,341 | 22,772,060 | 13,558,597 | 119,178,263 |
|  | % | 100.00 | 39.23 | 14.97 | 0.044 | 9.90 | 5.89 | 51.80 |
| *V.* sp. *C74* | Length | 106,485,303 | 33,213,526 | 19,071,335 | 121,799 | 29,944,169 | 9,725,844 | 50,824,528 |
|  | % | 100.00 | 31.19 | 17.91 | 0.114 | 28.12 | 9.13 | 47.73 |
| *V. stellata* | Length | 122,164,794 | 30,638,934 | 14,026,790 | 116,901 | 4,766,267 | 10,921,529 | 42,501,519 |
|  | % | 100.00 | 25.08 | 11.48 | 0.096 | 3.90 | 8.94 | 34.79 |
| *Mo.* sp.*C141* | Length | 60,466,283 | 3,930,511 | 2,293,929 | 21,529 | 13,422,493 | 11,536,668 | 23,838,835 |
|  | % | 100.00 | 6.50 | 3.79 | 0.036 | 22.20 | 19.08 | 39.43 |
| *Mo.* sp. *C73* | Length | 59,696,957 | 3,106,333 | 2,038,963 | 6,355 | 13,407,948 | 5,702,067 | 19,848,369 |
|  | % | 100.00 | 5.20 | 3.42 | 0.011 | 22.46 | 9.56 | 33.25 |
| *Mi. gaditana B-31* | Length | 26,801,226 | 158,351 | 64,627 | 680 | 773,224 | 85,734 | 1,076,991 |
|  | % | 100.00 | 0.59 | 0.24 | 0.003 | 2.89 | 0.32 | 4.02 |
| *Mi. salina CCMP537* | Length | 25,384,482 | 86,779 | 87,689 | 761 | 247,117 | 6,293 | 492,836 |
|  | % | 100.00 | 0.34 | 0.35 | 0.003 | 0.97 | 0.02 | 1.94 |
| *N. oceanica CCMP1779* | Length | 28,743,569 | 471,865 | 771,215 | 1,094 | 452,302 | 209,527 | 1,648,863 |
|  | % | 100.00 | 1.64 | 2.68 | 0.004 | 1.57 | 0.73 | 5.74 |
| *N. oculate CCMP525* | Length | 31,578,931 | 596,669 | 1,793,083 | 881 | 567,924 | 321,606 | 2,732,510 |
|  | % | 100.00 | 1.89 | 5.68 | 0.003 | 1.80 | 1.02 | 8.65 |
| *N. granulate CCMP529* | Length | 28,890,477 | 479,063 | 1,047,255 | 1,667 | 541,943 | 47,963 | 1,814,695 |
|  | % | 100.00 | 1.66 | 3.62 | 0.006 | 1.88 | 0.17 | 6.28 |
| *E. siliculosus* | Length | 195,810,619 | 7,378,904 | 11,618,736 | 312,295 | 31,382,900 | 3,812,785 | 57,963,865 |
|  | % | 100.00 | 3.77 | 5.93 | 0.159 | 16.03 | 1.95 | 29.60 |
| *S. japonica* | Length | 545,386,008 | 53,161,566 | 72,385,621 | 1,410,324 | 132,032,877 | 38,741,604 | 279,585,309 |
|  | % | 100.00 | 9.75 | 13.27 | 0.259 | 24.21 | 7.10 | 51.26 |
| *P. tricornutum* | Length | 27,450,724 | 352,533 | 102,908 | 1,901 | 2,714,106 | 3,316 | 3,385,491 |
|  | % | 100.00 | 1.28 | 0.37 | 0.007 | 9.89 | 0.01 | 12.33 |
| *T. pseudonana* | Length | 32,437,365 | 381,282 | 475,552 | 2,436 | 1,379,822 | 135,594 | 2,437,658 |
|  | % | 100.00 | 1.18 | 1.47 | 0.008 | 4.25 | 0.42 | 7.51 |

Note: all the repetitive elements were re-predicted in this study using the same pipeline.

**Table S7** BUSCO analysis of genesets of *V. sp. H4302* and *V*. *stellata* and genesets of other closely related species

| **BUSCO Assessment** | **Geneset** | | | |
| --- | --- | --- | --- | --- |
|  | **Complete** | **Duplicated** | **Fragmented** | **Missing** |
| *V.* sp. H4302 | 91% | 62% | 1% | 8% |
| *V. stellata* | 94% | 1% | 1% | 5% |
| *V.* sp. *C74* | 100% | 1% | 0% | 0% |
| *Mo.* sp. *C73* | 100% | 1% | 0% | 0% |
| *Mo.* sp. *C141* | 100% | 1% | 0% | 0% |
| *Mi. gaditana* B-31 | 85% | 1% | 10% | 5% |
| *N. oceanica* CCMP1779 | 88% | 3% | 4% | 8% |
| *Mi. salina* CCMP53*7* | 39% | 2% | 10% | 51% |
| *N. oculata* CCMP525 | 29% | 2% | 16% | 55% |
| *N. granulata* CCMP529 | 43% | 1% | 13% | 44% |

Note: orthologs database is stramenopiles_odb10.

**Table S8** Key resources table

| RESOURCE | SOURCE | IDENTIFIER |
| --- | --- | --- |
| *Vischeria* sp. CAUP H4302 | This study | CNGBdb: CNP0000525 |
| *Vischeria stellata* SAG 33.83 | This study | CNGBdb: CNP0000525 |
| *Vischeria* sp. C74 | Yang et al. 2021 [2] | https://figshare.com/s/c4bf156c2764ba410c30 |
| *Monodopsis* sp. C141 | Yang et al. 2021 [2] | https://figshare.com/s/c4bf156c2764ba410c30 |
| *Monodopsis* sp. C73 | Yang et al. 2021 [2] | https://figshare.com/s/c4bf156c2764ba410c30 |
| *Microchloropsis salina* CCMP537* | Wang et al. 2014 [1] | NCBI: PRJNA62503 |
| *Nannochloropsis oculata CCMP525** | Wang et al. 2014 [1] | NCBI: PRJNA65107 |
| *Nannochloropsis granulata* CCMP529* | Wang et al. 2014 [1] | NCBI: PRJNA65111 |
| *Microchloropsis gaditana* B31* | Corteggiani Carpinelli et al. 2014 [3] | http://www.nannochloropsis.org/page/ftp |
| *Nannochloropsis oceanica* CCMP1779* | Vieler et al. 2012 [4] | www.bmb.msu.edu/nannochloropsis.html |
| *Saccharina japonica** | Ye et al. 2015 [5] | NCBI: PRJNA272647 |
| *Ectocarpus siliculosus** | Cock et al. 2010 [6] | NCBI: PRJEA42625 |
| *Thalassiosira pseudonana** | Armbrust et al. 2004 [7] | NCBI: PRJNA191 |
| *Phaeodactylum tricornutum** | Bowler et al. 2008 [8] | NCBI: PRJNA13152 |

Note: *, the reference species used in Homology-based gene prediction.

**Table S9** Significant enriched KEGG pathways for lineage specific genes of Eustigmatophyceae

| **#Pathway** | **GN^*^** | **Adjust P** | **Level 2 label** |
| --- | --- | --- | --- |
| Biosynthesis of unsaturated fatty acids | 25 | 4.82E-08 | Lipid metabolism |
| Fatty acid elongation | 20 | 6.64E-08 | Lipid metabolism |
| ABC transporters | 31 | 1.27E-07 | Membrane transport |
| Fatty acid metabolism | 27 | 9.15E-05 | Global and overview maps |
| Biosynthesis of secondary metabolites | 116 | 1.71E-04 | Global and overview maps |
| beta-Alanine metabolism | 10 | 9.26E-04 | Metabolism of other amino acids |
| Steroid biosynthesis | 11 | 9.26E-04 | Lipid metabolism |
| Glycerophospholipid metabolism | 22 | 9.26E-04 | Lipid metabolism |
| Brassinosteroid biosynthesis | 4 | 1.29E-03 | Metabolism of terpenoids and polyketides |
| Butanoate metabolism | 12 | 2.42E-03 | Carbohydrate metabolism |
| Cutin, suberine and wax biosynthesis | 5 | 8.13E-03 | Lipid metabolism |
| Taurine and hypotaurine metabolism | 4 | 1.14E-02 | Metabolism of other amino acids |
| Glycerolipid metabolism | 18 | 1.45E-02 | Lipid metabolism |
| Vancomycin resistance | 2 | 3.37E-02 | Drug resistance: antimicrobial |
| Biotin metabolism | 8 | 3.37E-02 | Metabolism of cofactors and vitamins |
| Vitamin B6 metabolism | 7 | 3.71E-02 | Metabolism of cofactors and vitamins |
| Lysine biosynthesis | 7 | 4.94E-02 | Amino acid metabolism |

*: GN, Gene Number

**Table S10** Top 20 enriched KEGG pathways for lineage specific genes of *V.* sp. H4302

| #Pathway | GN | Adjust P | Level 2 label |
| --- | --- | --- | --- |
| Photosynthesis | 11 | 0.000151 | Energy metabolism |
| Selenocompound metabolism | 6 | 0.000514 | Metabolism of other amino acids |
| Ribosome | 14 | 0.003947 | Translation |
| Pyrimidine metabolism | 10 | 0.004179 | Nucleotide metabolism |
| RNA polymerase | 6 | 0.007967 | Transcription |
| ABC transporters | 6 | 0.062032 | Membrane transport |
| Other types of O-glycan biosynthesis | 3 | 0.152996 | Glycan biosynthesis and metabolism |
| One carbon pool by folate | 2 | 0.236068 | Metabolism of cofactors and vitamins |
| Oxidative phosphorylation | 5 | 0.236068 | Energy metabolism |
| Nitrogen metabolism | 2 | 0.24614 | Energy metabolism |
| Purine metabolism | 6 | 0.370832 | Nucleotide metabolism |
| Homologous recombination | 3 | 0.370832 | Replication and repair |
| Arginine biosynthesis | 2 | 0.386069 | Amino acid metabolism |
| Fatty acid degradation | 2 | 0.386069 | Lipid metabolism |
| Carotenoid biosynthesis | 2 | 0.458136 | Metabolism of terpenoids and polyketides |
| Fatty acid biosynthesis | 2 | 0.502438 | Lipid metabolism |
| Mismatch repair | 2 | 0.502482 | Replication and repair |
| RNA transport | 5 | 0.525676 | Translation |
| Proteasome | 2 | 0.555331 | Folding, sorting and degradation |
| 2-Oxocarboxylic acid metabolism | 2 | 0.565204 | Global and overview maps |

**Table S11** Statistics of transcriptome data mapped to *V.* sp. H4302 genome

| **Sample** | **High Quality Reads** | **Total Mapping Ratio** | **Uniquely MappingRatio** |
| --- | --- | --- | --- |
| NR-0h | 29,521,586 | 84.12% | 68.78% |
| NR-48h | 29,606,208 | 83.75% | 69.75% |
| NR-6d | 29,578,118 | 83.64% | 67.46% |
| NR-15d | 29,623,730 | 83.97% | 69.36% |
| NF-48h | 29,575,886 | 83.50% | 69.10% |
| NF-6d | 30,409,462 | 81.53% | 67.35% |
| NF_15d | 30,854,642 | 84.22% | 71.20% |
| NRC-24h | 29,416,876 | 84.45% | 69.16% |
| NRC-48h | 29,612,550 | 83.64% | 67.77% |
| NRC-96h | 29,908,300 | 77.59% | 59.09% |
| PF-48h | 29,497,576 | 84.23% | 70.50% |
| SF-48h | 29,454,336 | 83.81% | 68.83% |

# Reference

1. Wang D, Ning K, Li J, Hu J, Han D, Wang H, Zeng X, Jing X, Zhou Q, Su X *et al*: **Nannochloropsis genomes reveal evolution of microalgal oleaginous traits**. *PLoS Genet* 2014, **10**(1):e1004094.

2. Yang HP, Wenzel M, Hauser DA, Nelson JM, Xu X, Elias M, Li FW: **Monodopsis and Vischeria Genomes Shed New Light on the Biology of Eustigmatophyte Algae**. *Genome Biol Evol* 2021, **13**(11).

3. Corteggiani Carpinelli E, Telatin A, Vitulo N, Forcato C, D'Angelo M, Schiavon R, Vezzi A, Giacometti GM, Morosinotto T, Valle G: **Chromosome scale genome assembly and transcriptome profiling of Nannochloropsis gaditana in nitrogen depletion**. *Mol Plant* 2014, **7**(2):323-335.

4. Vieler A, Wu G, Tsai CH, Bullard B, Cornish AJ, Harvey C, Reca IB, Thornburg C, Achawanantakun R, Buehl CJ *et al*: **Genome, functional gene annotation, and nuclear transformation of the heterokont oleaginous alga Nannochloropsis oceanica CCMP1779**. *PLoS Genet* 2012, **8**(11):e1003064.

5. Ye NH, Zhang XW, Miao M, Fan X, Zheng Y, Xu D, Wang JF, Zhou L, Wang DS, Gao Y *et al*: **Saccharina genomes provide novel insight into kelp biology**. *Nature Communications* 2015, **6**.

6. Cock JM, Sterck L, Rouze P, Scornet D, Allen AE, Amoutzias G, Anthouard V, Artiguenave F, Aury JM, Badger JH *et al*: **The Ectocarpus genome and the independent evolution of multicellularity in brown algae**. *Nature* 2010, **465**(7298):617-621.

7. Armbrust EV, Berges JA, Bowler C, Green BR, Martinez D, Putnam NH, Zhou S, Allen AE, Apt KE, Bechner M *et al*: **The genome of the diatom Thalassiosira pseudonana: ecology, evolution, and metabolism**. *Science* 2004, **306**(5693):79-86.

8. Bowler C, Allen AE, Badger JH, Grimwood J, Jabbari K, Kuo A, Maheswari U, Martens C, Maumus F, Otillar RP *et al*: **The Phaeodactylum genome reveals the evolutionary history of diatom genomes**. *Nature* 2008, **456**(7219):239-244.
